# Polar lipidomic profile shows *Chlorococcum amblystomatis* as a promising source of value-added lipids

**DOI:** 10.1038/s41598-021-83455-y

**Published:** 2021-02-23

**Authors:** Tiago A. Conde, Daniela Couto, Tânia Melo, Margarida Costa, Joana Silva, M. Rosário Domingues, Pedro Domingues

**Affiliations:** 1 Department of Chemistry, Mass Spectrometry Centre, LAQV REQUIMTE, University of Aveiro, Santiago University Campus, 3810-193 Aveiro, Portugal; 2 Department of Chemistry, CESAM-Centre for Environmental and Marine Studies, University of Aveiro, Santiago University Campus, 3810-193 Aveiro, Portugal; 3 R&D Department, Allmicroalgae Natural Products S.A., Rua 25 de Abril 19, 2445-287 Pataias, Portugal

**Keywords:** Lipidomics, Lipids

## Abstract

There is a growing trend to explore microalgae as an alternative resource for the food, feed, pharmaceutical, cosmetic and fuel industry. Moreover, the polar lipidome of microalgae is interesting because of the reports of bioactive polar lipids which could foster new applications for microalgae. In this work, we identified for the first time the *Chlorococcum amblystomatis* lipidome using hydrophilic interaction liquid chromatography-high resolution electrospray ionization- tandem mass spectrometry (HILIC–HR–ESI–MS/MS). The *Chlorococcum amblystomatis* strain had a lipid content of 20.77% and the fatty acid profile, determined by gas chromatography-mass spectrometry, has shown that this microalga contains high amounts of omega-3 polyunsaturated fatty acids (PUFAs). The lipidome identified included 245 molecular ions and 350 lipid species comprising 15 different classes of glycolipids (6), phospholipids (7) and betaine lipids (2). Of these, 157 lipid species and the main lipid species of each class were esterified with omega-3 PUFAs. The lipid extract has shown antioxidant activity and anti-inflammatory potential. Lipid extracts also had low values of atherogenic (0.54) and thrombogenic index (0.27). In conclusion, the lipid extracts of *Chlorococcum amblystomatis* have been found to be a source of lipids rich in omega-3 PUFAs for of great value for the food, feed, cosmetic, nutraceutical and pharmaceutical industries.

## Introduction

Reducing the impact of climate change is one of the main challenges of this decade, which has increased the demand to move to more environmental and sustainable resources^1,2^. Microalgae represent a sustainable^3,4^ and versatile^5,6^ resource with exploitation potential for different industries (for example food, feed, nutraceutical, cosmetic, pharmaceutical)^7^. These unicellular organisms are found not only in aquatic systems but also in terrestrial systems, with a wide range of catalogued species^8,9^. Microalgae are valued for their photosynthetic efficiency, low water requirement, fast growth and high biomass production^10^, which allows efficient biomass production with lower resource consumption that meet industry’s needs. Also, microalgae grow with greater photosynthetic efficiency than higher plants and do not require arable land to be cultivated^11^. Microalgae have been used for different applications, namely, as direct food, as food nutrients, for animal feed, as sources of bioactive products for the pharmaceutical industry, and as a source of lipids for the production of biodiesel^5^. For example, microalgae can consume nutrients that are in excess in the agri-food industry waste streams, such as nitrogen and phosphorous^12^.. In terms of nutrients, they are a rich source of essential amino acids, vitamins, minerals, pigments and lipids, namely polyunsaturated fatty acids (PUFAs)^13^. They are considered a promising source of bioactive components due to their antioxidant, anti-inflammatory, anti-obesity, anti-tumour, antiviral and anti-bacterial properties^14^. Among the bioactive compounds described, phospholipids (PL) seem to be interesting ingredients as the main vector of omega-3 fatty acids (FA)^15^ and glycolipids (GL) as promising bioactive phytochemicals^16^. There is a large body of evidence suggesting that omega-3 PUFAs esterified in polar lipids have important physiological properties. For example, PL and GL containing omega-3 FA, were associated with antioxidant and anti-inflammatory properties, and with cardiovascular protection^17^ and also that PLs esterified in omega-3 PUFAs are excellent delivery vehicles^18,19^. In addition, GLs with PUFAs have shown anti-inflammatory potential with nitric oxide release inhibitory activity^20,21^ while DGDGs and SQDGs from algae showed chemotherapeutic potential^22^ and SQDG esterified in EPA also displays an anti-proliferative effect^23^. Very recent studies have focused on the identification of polar lipids from the most commercially used microalgae, such as *Chlorella* sp. and *Chlorella vulgaris*^24,25^, *Nannochloropsis oceanica* and *Nannochloropsis oculata*^26,27^, and *Phaeodactylum tricornutum*^28^. However, polar lipids are scarcely studied compared to other ingredients derived from microalgae, an even larger number of microalgae lipidome remains unknown, unexplored or poorly studied, hampering the full biotechnological exploitation of this resource.

*Chlorococcum* sp. is a green microalga which belongs to the phylum Chlorophyta, class Chlorophyceae, order Chlorococcaceae and family Chlorococcaceae, and it can be cultivated under photoautotrophic and mixotrophic conditions^29^. The biotechnological potential of this microalga remains poorly explored^29^. *Chlorococcum* sp. has been described as a useful tool in carbon sequestration, lipid flocculation in wastewater^30^ and carotenoid production^31^, a good source for the production of biodiesel^32^ and useful for the food industry^33,34^. The production of lutein from *Chlorococcum citriforme* has already been patented^35^. Regarding its biochemical composition, a total biomass dry weight content of proteins of 13.08–40%^32,36^, carbohydrates of 3.42–40%^32,36^, chlorophylls of 0.4–1.5%^32^, carotenoids of 3.2–12.6 mg/g^37^ and lipids of 8.71–32.3%^32,36,38–40^ has been described. In addition to its high lipid content, the FA composition of the *Chlorococcum* strains studied to date, included good amounts of omega-3 fatty acids^33^ suggesting the possibility of added-value lipids that needs to be explored.

The interest of knowing the lipidome of green microalgae, either to optimize the production of biodiesel or to produce lipids with added value, has led to the complete profiling of several microalgae^16,27^. As such, the complete lipidome has been reported for certain green microalgae belonging to the phylum Chlorophyta, such as *Haematococcus* sp.^27^, *Chlamydomonas* sp.^41^, *Scenedesmus* sp.^24^, *Chlorella* sp.^42^ and *Chlorella kessleri*^43^. Microalgal lipids have received much attention due to their high abundance of omega-3 and omega-6 PUFAs, and, more recently, due to their polar lipids content (e.g. monogalactosyldiacylglycerol [MGDG], digalactosyldiacylglycerol [DGDGs]), in which the PUFAs are esterified. These glycolipids are plastid lipids, therefore abundant in green microalgae. Also, these lipids were reported to have desired bioactive properties, such as antibacterial, antioxidant and anti-inflammatory activities^16^. PUFAs were already described in *Chlorococcum* sp*.*^32,33,38,39^, however, the polar lipidome of this microalga remains to be described, preventing its full exploitation. Thus, to fill this gap, in the present work, we have analysed and characterized in detail the polar lipidome of *Chlorococcum amblystomatis*, cultivated in autotrophic conditions, using a lipidomic approach based on the hydrophilic interaction liquid chromatography-electrospray ionization-high resolution mass spectrometry (HILIC–HR–ESI–MS). We also explored the bioactive potential of lipid extracts of *Chlorococcum amblystomatis* to inhibit COX-2 activity and its antioxidant properties, using commercial kits, to promote valorisation of this microalga as a source of bioactive lipids for the food, feed, cosmetics or pharmaceutical industries.

## Results

### Total lipid content and fatty acid composition of *Chlorococcum amblystomatis*

The lipid extracts of *Chlorococcum amblystomatis* corresponded to an average yield of 20.77 ± 0.57% of microalgal biomass. The fatty acid composition of *Chlorococcum amblystomatis* identified by GC–MS showed that C16:0 was the most abundant FA (23.0 ± 0.8%), followed by C18:3 *n*-3 (19.4 ± 0.6%), C18:0 (13.9 ± 2.3%), C16:4 *n*-3 (11.1 ± 0.6%), C20:5 *n*-3 (8.9 ± 0.6%) and C16:1 *n*-9 (7.5 ± 0.3%) (Table 1). The remaining identified FAs had relative abundances of less than 5%. This microalga had a total omega-3 fatty acid content of 43.2%.

**Table 1:** Fatty acid profile identified in the total lipid extract of *Chlorococcum amblystomatis* by GC–MS.

Fatty acids	Relative abundance (%) ± SD
C12:0	0.1 ± 0.0
C14:0	1.5 ± 0.2
C15:0-iso	0.0 ± 0.0
C15:0	0.2 ± 0.0
C16:0	23.0 ± 0.8
C16:1 Δ^9^	7.5 ± 0.3
C16:1 Δ^11^	0.1 ± 0.0
C16:2 Δ^7.10^ (*n*−6)	0.3 ± 0.0
C16:3 Δ^4,7,10^ (*n*−6)	0.6 ± 0.1
C17:1	0.1 ± 0.0
C16:3 Δ^7,10,13^ (*n*−3)	0.6 ± 0.1
C16:4 Δ^4,7,10,13^ (*n*−3)	11.1 ± 0.6
C18:0	13.9 ± 2.3
C18:1 Δ^9^	1.6 ± 0.1
C18:1 Δ^11^	3.6 ± 0.2
C18:2 Δ^9,12^ (*n*−6)	2.3 ± 0.2
C18:3 Δ^6,9,12^ (*n*−6)	1.0 ± 0.1
C18:3 Δ^9,12,15^ (*n*−3)	19.4 ± 0.6
C18:4 Δ^6,9,12,15^ (*n*−3)	3.2 ± 0.2
C20:0	0.2 ± 0.0
C20:4 Δ^5,8,11,14^ (*n*−6)	1.1 ± 0.1
C20:5 Δ^5,8,11,14,17^ (*n*−3)	8.9 ± 0.6
Σ SFA	38.8
Σ MUFAs	12.9
Σ PUFAs	48.4
Σ (n−3)	43.2
Σ (n−6)	5.2
n−6/n−3 ratio	0.1
AI	0.5
TI	0.3
(h/H)	1.4

Values are expressed in relative abundance (%) and represent the mean of five analytical samples ± standard deviation (SD).

*SFA* saturated fatty acids, *MUFAs* monounsaturated fatty acids, *PUFAs* polyunsaturated fatty acids, *AI* atherosclerotic index, *TI* thrombogenic index, *(h/H)* (hypocholesterolemic/hypercholesterolemic) ratio.

To assess the potential health benefits of *Chlorococcum amblystomatis*, the atherogenic (AI), thrombogenic (TI), hypocholesterolemic/hypercholesterolemic (h/H) and the PUFA *n*-6/ PUFA *n*-3 indices were calculated. The AI and TI of the lipid extracts were 0.5 and 0.3, respectively, while the h/H ratio was 1.4 and the *n*-6/*n*-3 ratio was 0.1.

### Polar lipid composition of *Chlorococcum amblystomatis*

The polar lipid profile was revealed by hydrophilic interaction liquid chromatography-high resolution electrospray mass spectrometry (HILIC–HR–ESI–MS and HILIC–HR–ESI–MS/MS) (Supplementary file S1). Using this approach, a total of 245 molecular ions, with a minimum of 350 molecular species, belonging to 15 classes of lipids were identified. Of these, 101 molecular ions were glycolipids (GL), 54 molecular ions were betaine lipids (BL), 89 molecular ions were phospholipids (PL), and 1 molecular ion was an inositol phosphoceramide (PI-Cer) (Tables 2 and 3). Figures explaining how the MS/MS data were interpreted can be found in the Supplementary Figures S1–S13. The *m/z* of the fragment ions used to identify each molecular ion and each molecular species can be found in Supplementary Table S1 and S2.

**Table 2:** Polar lipid classes identified in the total lipid extract of *Chlorococcum amblystomatis* by HILIC–HR–ESI–MS and MS/MS.

Lipid classes	*Chlorococcum amblystomatis*		
	Molecular ions number	Lipid species number	Major species
**Glycolipids**	101	158	–
MGDG	30	47	MGDG(34:7)
MGMG	11	11	MGMG(16:4)
DGDG	27	48	DGDG(34:3)
DGMG	11	11	DGMG(18:3)
SQDG	21	40	SQDG(32:1)
SQMG	1	1	SQMG(16:0)
**Phospholipids**	89	107	–
LPC	11	11	LPC(16:1)
PC	32	39	PC(34:2)
LPE	9	9	LPE(16:0)
PE	13	14	PE(34:4)
PG	22	32	PG(34:3)
PI	2	2	PI(32:1)
**Betaine lipids**	54	84	
DGTS	38	68	DGTS(34:4)
MGTS	16	16	MGTS(18:4)
**Sphingolipids**	1	1	
PI-Cer	1	1	PI-Cer(d18:1/14:0)
Total	245	350	–

The total number of molecular ions, lipid species identified in each class and lipid species per class is shown.

*MGDG* monogalactosyldiacylglycerol, *MGMG* monogalactosylmonoacylglycerol, *DGDG* digalactosyldiacylglycerol, *DGMG* digalactosylmonoacylglycerol, *SQDG* sulfoquinovosyl diacylglycerol, *SQMG* sulfoquinovosyl monoacylglycerol, *LPC* lysophosphatidylcholine, *PC* phosphatidylcholine, *LPE* lysophosphatidylethanolamine, *PE* phosphatidylethanolamine, *PG* phosphatidylglycerol, *PI* phosphatidylinositol, *DGTS* diacylglycerol-trimethylhomoserine, *MGTS* monoacylglycerol-trimethylhomoserine, *PI-Cer* inositol phosphoceramide.

**Table 3:** Phospholipid species identified in the total lipid extract of *Chlorococcum amblystomatis* by HILIC–HR–ESI–MS and MS/MS.

Lipid species (C:N)	Calculated *m/z*	Observed *m/z*	Error (ppm)	Fatty acyl chains (C:N)	Formula
**LPC identified as [M+H]**^**+**^					
LPC14:0)	468.3090	468.3086	− 0.9	**	C_22_H_47_NO_7_P
LPC(16:0)	496.3403	496.3404	0.2	**	C_24_H_51_NO_7_P
LPC(16:1)	494.3247	494.3244	− 0.5	16:1	C_24_H_49_NO_7_P
LPC(16:2)	492.3090	492.3083	− 1.5	**	C_24_H_47_NO_7_P
LPC(18:1)	522.3560	522.3561	0.3	18:1	C_26_H_53_NO_7_P
LPC(18:2)	520.3403	520.3402	− 0.2	18:2	C_26_H_51_NO_7_P
LPC(18:3)	518.3247	518.3233	− 2.6	*	C_26_H_49_NO_7_P
LPC(18:4)	516.3090	516.3067	− 4.5	*	C_26_H_47_NO_7_P
LPC(20:3)	546.3560	546.3560	0.1	**	C_28_H_53_NO_7_P
LPC(20:4)	544.3403	544.3398	− 0.9	**	C_28_H_51_NO_7_P
LPC(20:5)	542.3247	542.3241	− 1.0	*	C_28_H_49_NO_7_P
**PC identified as [M+H]**^**+**^					
PC(28:1)	676.4917	676.4905	− 1.8	*	C_36_H_71_NO_8_P
PC(30:0)	706.5387	706.5371	− 2.2	**	C_38_H_77_NO_8_P
PC(30:1)	704.5230	704.5250	2.8	16:1–14:0	C_38_H_75_NO_8_P
PC(30:3)	700.4917	700.4895	− 3.2	*	C_38_H_71_NO_8_P
PC(32:1)	732.5543	732.5539	− 0.6	16:1–16:0	C_40_H_79_NO_8_P
PC(32:2)	730.5387	730.5384	− 0.4	16:1–16:1	C_40_H_77_NO_8_P
PC(32:3)	728.5230	728.5230	0.0	16:1–16:2	C_40_H_75_NO_8_P
PC(32:4)	726.5074	726.5058	− 2.2	*	C_40_H_73_NO_8_P
PC(32:5)	724.4917	724.4908	− 1.3	**	C_40_H_71_NO_8_P
PC(34:1)	760.5856	760.5842	− 1.9	16:0–18:1 and 16:1–18:0	C_42_H_83_NO_8_P
PC(34:2)	758.5700	758.5696	− 0.5	16:1–18:1 and 16:0–18:2	C_42_H_81_NO_8_P
PC(34:3)	756.5543	756.5542	− 0.2	16:1–18:2	C_42_H_79_NO_8_P
PC(34:4)	754.5387	754.5371	− 2.1	16:1–18:3 and 16:2–18:2	C_42_H_77_NO_8_P
PC(34:5)	752.5230	752.5210	− 2.7	*	C_42_H_75_NO_8_P
PC(34:6)	750.5074	750.5064	− 1.3	*	C_42_H_73_NO_8_P
PC(34:7)	748.4917	748.4899	− 2.4	**	C_42_H_71_NO_8_P
PC(36:2)	786.6013	786.6004	− 1.1	**	C_44_H_85_NO_8_P
PC(36:3)	784.5856	784.5850	− 0.8	18:1–18:2	C_44_H_83_NO_8_P
PC(36:4)	782.5700	782.5682	− 2.3	18:2–18:2 and 16:0–20:4	C_44_H_81_NO_8_P
PC(36:5)	780.5543	780.5528	− 2.0	18:3–18:2, 16:0–20:5 and 16:1–20:4	C_44_H_79_NO_8_P
PC(36:6)	778.5387	778.5379	− 1.0	16:1–20:5	C_44_H_77_NO_8_P
PC(36:7)	776.5230	776.5212	− 2.4	*	C_44_H_75_NO_8_P
PC(38:5)	808.5856	808.5843	− 1.6	18:1–20:4	C_46_H_83_NO_8_P
PC(38:6)	806.5700	806.5690	− 1.2	18:2–20:4 and 18:1–20:5	C_46_H_81_NO_8_P
PC(38:7)	804.5543	804.5525	− 2.3	18:2–20:5	C_46_H_79_NO_8_P
PC(38:8)	802.5387	802.5351	− 4.5	**	C_46_H_77_NO_8_P
PC(38:9)	800.5230	800.5204	− 3.3	*	C_46_H_75_NO_8_P
PC(40:10)	826.5387	826.5369	− 2.2	*	C_48_H_77_NO_8_P
PC(40:5)	836.6169	836.6180	1.3	*	C_48_H_87_NO_8_P
PC(40:7)	832.5856	832.5831	− 3.0	**	C_48_H_83_NO_8_P
PC(40:8)	830.5700	830.5674	− 3.1	*	C_48_H_81_NO_8_P
PC(40:9)	828.5543	828.5520	− 2.8	*	C_48_H_79_NO_8_P
**LPE identified as [M+H]**^**+**^					
LPE(14:0)	426.2621	426.2615	− 1.3	**	C_19_H_41_NO_7_P
LPE(16:0)	454.2934	454.2928	− 1.2	16:0	C_21_H_45_NO_7_P
LPE(16:1)	452.2777	452.2774	− 0.7	*	C_21_H_43_NO_7_P
LPE(16:4)	446.2308	446.2303	− 1t.0	**	C_21_H_37_NO_7_P
LPE(18:1)	480.3090	480.3092	0.4	18:1	C_23_H_47_NO_7_P
LPE(18:2)	478.2934	478.2926	− 1.6	**	C_23_H_45_NO_7_P
LPE(18:3)	476.2777	476.2774	− 0.7	*	C_23_H_43_NO_7_P
LPE(18:4)	474.2621	474.2618	− 0.6	18:4	C_23_H_41_NO_7_P
LPE(20:5)	500.2777	500.2781	0.8	**	C_25_H_43_NO_7_P
**PE identified as [M+H]**^**+**^					
PE(30:1)	662.4761	662.4754	− 1.0	14:0–16:1	C_35_H_69_NO_8_P
PE(30:3)	658.4448	658.4422	− 3.9	*	C_35_H_65_NO_8_P
PE(32:1)	690.5074	690.5058	− 2.3	*	C_37_H_73_NO_8_P
PE(32:2)	688.4917	688.4925	1.1	16:1–16:1	C_37_H_71_O_8_NP
PE(32:4)	684.4604	684.4593	− 1.7	16:4–16:0	C_37_H_67_NO_8_P
PE(34:1)	718.5387	718.5353	− 4.7	16:0–18:1	C_39_H_77_NO_8_P
PE(34:2)	716.5230	716.5219	− 1.6	16:1–18:1 and 16:0–18:2	C_39_H_75_NO_8_P
PE(34:3)	714.5074	714.5044	− 4.2	*	C_39_H_73_O_8_NP
PE(34:4)	712.4917	712.4901	− 2.3	16:0–18:4	C_39_H_71_NO_8_P
PE(34:5)	710.4761	710.4754	− 1.0	16:1–18:4	C_39_H_69_NO_8_P
PE(36:2)	744.5543	744.5535	− 1.1	18:1–18:1	C_41_H_79_O_8_NP
PE(36:5)	738.5074	738.5075	0.2	18:4–18:1	C_41_H_73_O_8_NP
PE(36:6)	736.4917	736.4917	0.0	*	C_41_H_71_NO_8_P
**PG identified as [M−H]**^**−**^					
PG(30:0)	693.4707	693.4700	− 1.0	14:0–16:0	C_36_H_70_O_10_P
PG(30:1)	691.4550	691.4545	− 0.7	**	C_36_H_68_O_10_P
PG(32:0)	721.5020	721.5026	0.8	**	C_38_H_74_O_10_P
PG(32:1)	719.4863	719.4866	0.4	16:1–16:0 and 14:0–18:1	C_38_H_72_O_10_P
PG(32:2)	717.4707	717.4703	− 0.6	16:1–16:1	C_38_H_70_O_10_P
PG(34:1)	747.5176	747.5181	0.7	16:0–18:1	C_40_H_76_O_10_P
PG(34:2)	745.5020	745.5011	− 1.2	**	C_40_H_74_O_10_P
PG(34:3)	743.4867	743.4868	0.1	16:0–18:3	C_40_H_72_O_10_P
PG(34:4)	741.4707	741.4711	0.5	16:0–18:4 and 16:1–18:3	C_40_H_70_O_10_P
PG(34:5)	739.4550	739.4534	− 2.2	14:0–20:5 and 16:2–18:3	C_40_H_68_O_10_P
PG(36:2)	773.5333	773.5337	0.5	18:1–18:1	C_42_H_78_O_10_P
PG(36:5)	767.4864	767.4863	− 0.1	16:0–20:5	C_42_H_72_O_10_P
PG(36:6)	765.4707	765.4715	1.0	16:1–20:5	C_42_H_70_O_10_P
PG(38:5)	795.5176	795.5197	2.6	**	C_44_H_76_O_10_P
PG(32:2-OH)	733.4656	733.4653	− 0.4	16:0-OH-16:2	C_38_H_70_O_11_P
PG(34:1-OH)	763.5125	763.5115	− 1.3	18:0-OH-16:1	C_40_H_76_O_11_P
PG(34:2-OH)	761.4969	761.4971	0.2	18:0-OH-16:2	C_40_H_74_O_11_P
PG(34:3-OH)	759.4812	759.4808	− 0.5	18:3-OH-16:0, 18:2-OH-16:1, 18:1-OH-16:2 and 18:0-OH-16:3	C_40_H_72_O_11_P
PG(34:4-OH)	757.4656	757.4653	− 0.4	18:4-OH-16:0, 18:3-OH-16:1 and 18:2-OH-16:2	C_40_H_70_O_11_P
PG(34:5-OH)	755.4499	755.4480	− 2.5	18:3-OH-16:2 and 18:4-OH-16:1	C_40_H_68_O_11_P
PG(36:5-OH)	783.4812	783.4828	2.0	16:0-OH-20:5 and 20:5-OH-16:0	C_42_H_72_O_11_P
PG(36:6-OH)	781.4656	781.4665	1.1	20:5-OH-16:1	C_42_H_70_O_11_P
**PI identified as [M−H]**^**−**^					
PI(32:1)	807.5024	807.5018	− 0.7	16:0–16:1	C_41_H_76_O_13_P
PI(34:1)	835.5318	835.5302	− 1.9	16:0–18:1	C_43_H_80_O_13_P
**PI-Cer identified as [M−H]**^**−**^					
PI-Cer(d18:1/14:0)	750.4921	750.4921	0.0	*	C_38_H_73_NO_11_P

*C* number of carbon atoms, *N* number of double bonds.

*Identified based on the polar head fragmentation, calculated mass, and retention time.

**Identified according to the calculated mass and the retention time.

**Figure 1:**
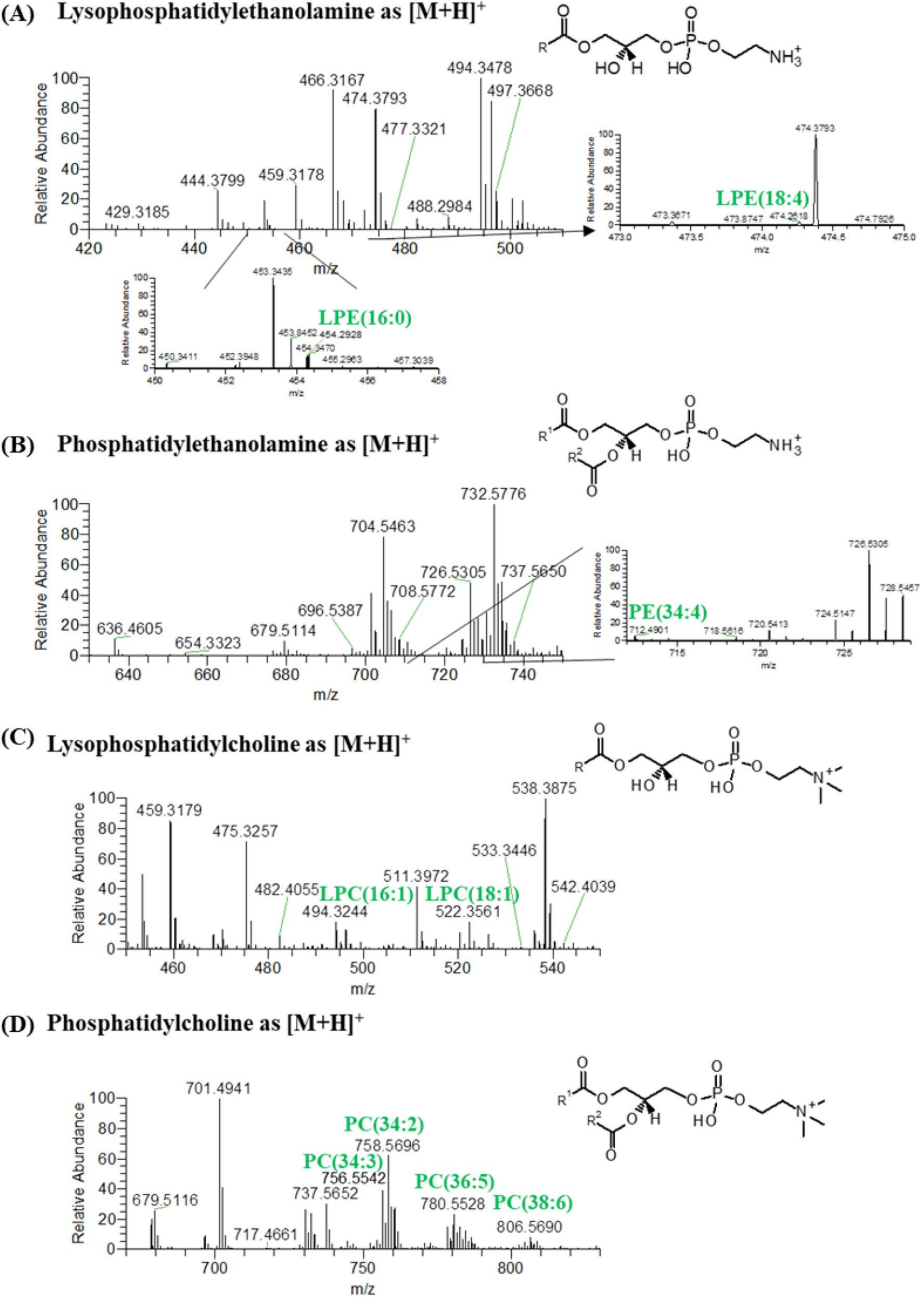
HILIC–HR–ESI–MS spectra of the classes of phospholipids (PL) identified in positive ion mode as [M+H]^+^. These classes were (**A**) lysophosphatidylethanolamine (LPE), (**B**) phosphatidylethanolamine (PE), (**C**) lysophosphatidylcholine (LPC) and (**D**) phosphatidylcholine (PC).

**Figure 2:**
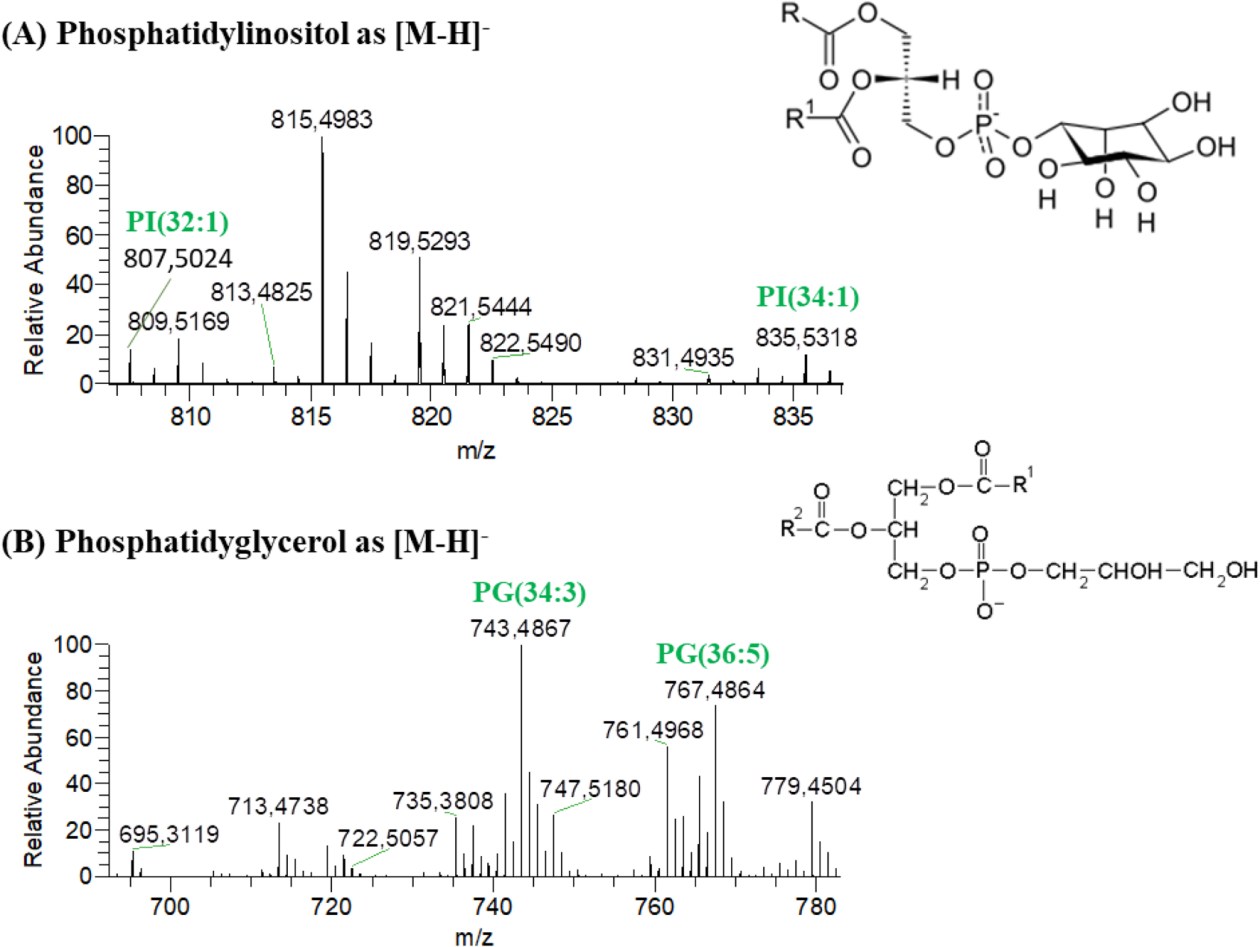
HILIC–HR–ESI–MS spectra of classes of phospholipids (PL) identified in negative ion mode as [M−H]^−^. These classes were (**A**) phosphatidylinositol (PI) and (**B**) phosphatidylglycerol (PG).

The PL classes identified included phosphatidylcholine (PC), lysophosphatidylcholine (LPC), phosphatidylethanolamine (PE) and lysophosphatidylethanolamine (LPE), identified in the HILIC–HR–ESI–MS analysis as the molecular ion [M+H]^+^ (Fig. 1); and phosphatidylglycerol (PG) and phosphatidylinositol (PI) identified as the negative molecular ion, [M−H]^−^ (Fig. 2). The only sphingolipid species found in *Chlorococcum amblystomatis* was a PI-Cer, which has been identified in the analysis on the negative ion mode. A total of 32 molecular ions of PC, 11 of LPC, 13 of PE, 9 of LPE, 22 of PG, 2 of PI and 1 of PI-Cer were recognized. Analysis of the MS/MS data allowed to recognize that each ion can correspond to several molecular species, with different combinations of fatty acyl chains, but with the same (C:N) (Supplementary Table S2). The molecular species identified per class were of 39 PC, 11 LPC, 14 PE, 9 LPE, 32 PG, 2 PI, and 1 PI-Cer.

As previously indicated, the identification of the lipid species was based on the mass accuracy, retention time and interpretation of MS/MS data. The interpretation of the MS/MS data of the molecular ions of the PC, LPC, PE and LPE species allowed to confirm their identity (Supplementary Table S2). This was done by confirming for each species the presence of the polar head groups, in particular by the presence of the product ion at *m/z* 184 (for PC and LPC) and fragment ions arising from the neutral loss of 141 Da (for PE and LPE)^16,44–48^. On the other hand, the complementary analysis of the molecular ions on the HILIC–HR–ESI–MS/MS spectra acquired in negative ion mode, observed as [M+CH_3_COO]^−^ ions for PC and LPC and [M−H]^−^ ions for PE and LPE, allowed to assign the fatty acyl composition. This was done by observing the presence of the RCOO^−^carboxylate fragment anions of the fatty acids esterified in the glycerol backbone^16,44–48^.

The HILIC–HR–ESI–MS/MS spectra of the negative molecular ions belonging to the PG, PI and PI-Cer classes allowed to confirm the presence of the corresponding polar heads by observing for each species the product ions at *m/z* 171 (for PG) and *m/z* 241 (for PI and PI-Cer), and identify the fatty acyl chains by the presence of RCOO^−^carboxylate anions (for PG and PI)^16,44–48^ (Supplementary Table S2). The only PI-Cer detected was identified by the accurate mass measurement, the retention time, and the presence of the polar head at *m/z* 241. The most abundant species in each phospholipid class were: PC(34:2) identified as PC(16:1–18:1) and PC(16:0–18:2); LPC(16:1); PE(34:4) identified as PE(16:0–18:4); LPE(16:0); PG(34:3) identified as PG(16:0–18:3); and PI(32:1) identified as PI(16:0–16:1).

Among the PG molecular ions identified, a few oxidized PG species were found (Table 3), the most abundant being PG(34:2-OH), identified as PG(18:0-OH-16:2). All the oxidized PG species were identified based on exact mass measurements, retention time and MS/MS spectra analysis (Supplementary Table S3). In the MS/MS spectra, the presence of mass shifts of + 16 Da relative to the [M−H]^−^ ions and of RCOO^−^carboxylate hydroxyl anions was indicative of the formation of hydroxy derivatives^49,50^. No product ions with oxidized polar head groups were observed ^45^.

**Figure 3:**
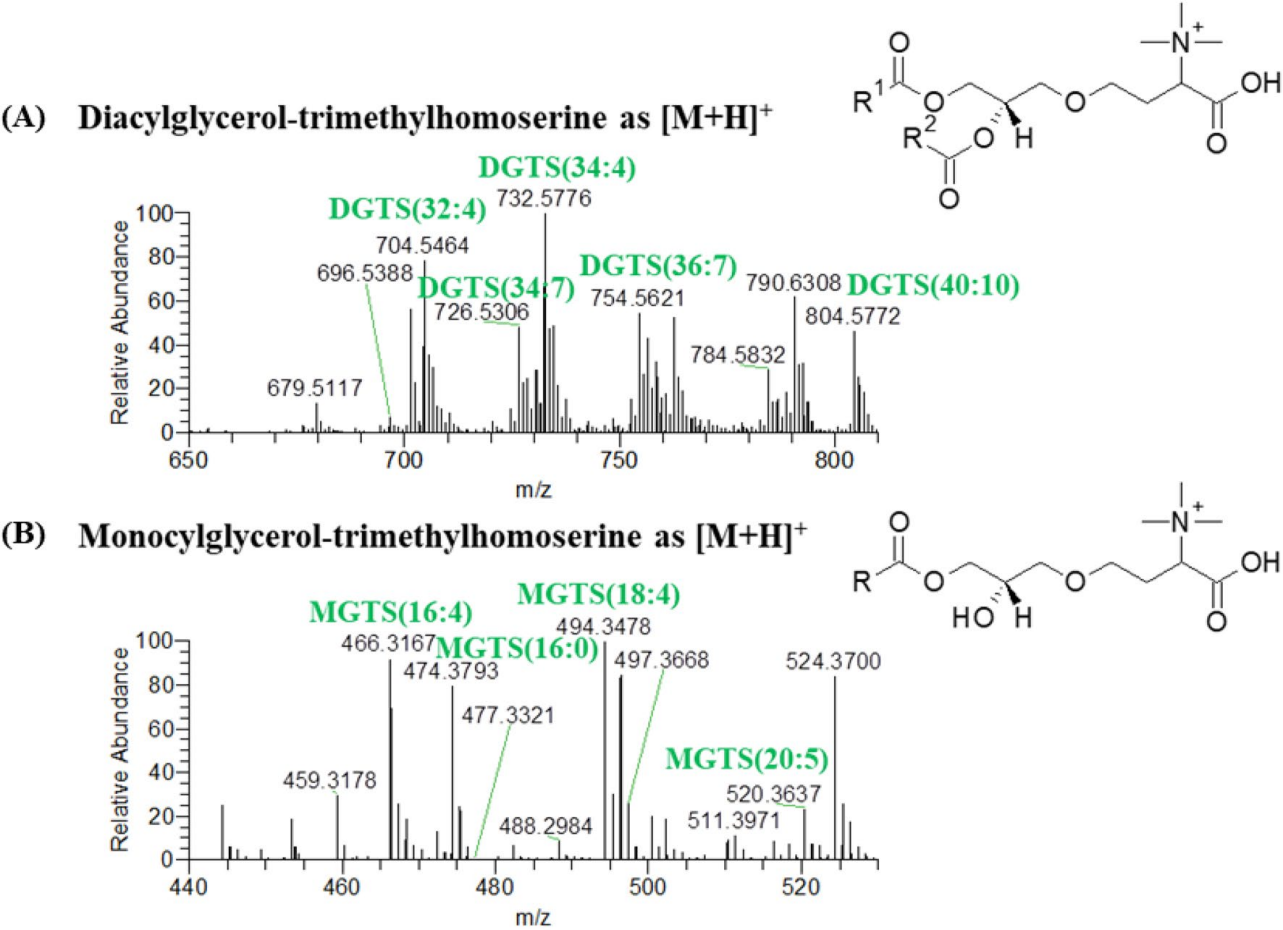
HILIC–HR–ESI–MS spectra of the classes of betaine lipids (BL) identified in positive ion mode as [M+H]^+^. These classes were (**A**) diacylglycerol-trimethylhomoserine (DGTS) and (**B**) monoacylglycerol-trimethylhomoserine (MGTS).

The betaine lipids identified in the HILIC–HR–ESI–MS data included the classes of monoacylglycerol-trimethylhomoserine (MGTS) and diacylglycerol-trimethylhomoserine (DGTS), and were identified in positive ion mode as [M+H]^+^ ions (Table 4, Fig. 3). A total of 38 molecular ions of DGTS and 16 molecular ions of MGTS were identified, corresponding to 68 molecular species of DGTS and 16 molecular species of MGTS. In the HILIC–HR–ESI–MS/MS spectra of MGTS and DGTS, as [M+H]^+^ ions, the presence of the polar head group in each species was confirmed by observation of the product ion at *m/z* 236^44,47^, and the fatty acyl composition was determined by observation of the fragment ions resulting from the neutral losses of the fatty acyl chains as an acid (–RCOOH) and ketene (–R=C=O) derivatives^44,47^ (Supplementary Table S2).

**Table 4:** Betaine lipids identified in the polar lipidome of *Chlorococcum amblystomatis* by HILIC–HR–ESI–MS and MS/MS.

Lipid species (C:N)	Calculated *m/z*	Observed *m/z*	Error (ppm)	Fatty acyl chains (C:N)	Formula
**MGTS identified as [M+H]**^**+**^					
MGTS(14:0)	446.3482	446.3479	− 0.6	14:0	C_24_H_48_O_6_N
MGTS(14:1)	444.3325	444.3323	− 0.5	14:1	C_24_H_46_O_6_N
MGTS(16:0)	474.3795	474.3793	− 0.4	16:0	C_26_H_52_O_6_N
MGTS(16:1)	472.3638	472.3635	− 0.7	16:1	C_26_H_50_O_6_N
MGTS(16:2)	470.3482	470.3485	0.7	16:2	C_26_H_48_O_6_N
MGTS(16:3)	468.3325	468.3335	2.1	**	C_26_H_46_O_6_N
MGTS(16:4)	466.3169	466.3167	− 0.4	16:4	C_26_H_44_O_6_N
MGTS(18:0)	502.4108	502.4115	1.5	18:0	C_28_H_56_O_6_N
MGTS(18:1)	500.3951	500.3948	− 0.6	18:1	C_28_H_54_O_6_N
MGTS(18:2)	498.3795	498.3789	− 1.1	**	C_28_H_52_O_6_N
MGTS(18:3)	496.3638	496.3636	− 0.4	**	C_28_H_50_O_6_N
MGTS(18:4)	494.3482	494.3478	− 0.7	18:4	C_28_H_48_O_6_N
MGTS(18:5)	492.3325	492.3318	− 1.5	18:5	C_28_H_46_O_6_N
MGTS(20:0)	530.4421	530.4402	− 3.5	20:0	C_30_H_60_O_6_N
MGTS(20:4)	522.3795	522.3798	0.6	**	C_30_H_52_O_6_N
MGTS(20:5)	520.3638	520.3637	− 0.2	20:5	C_30_H_50_O_6_N
**DGTS identified as [M+H]**^**+**^					
DGTS(28:1)	654.5309	654.5325	2.4	*	C_38_H_72_O_7_N
DGTS(30:0)	684.5778	684.5759	− 2.8	16:0–14:0	C_40_H_78_O_7_N
DGTS(30:1)	682.5622	682.5619	− 0.4	16:1–14:0	C_40_H_76_O_7_N
DGTS(30:2)	680.5465	680.5444	− 3.1	16:2–14:0	C_40_H_74_O_7_N
DGTS(30:3)	678.5309	678.5306	− 0.4	**	C_40_H_72_O_7_N
DGTS(30:4)	676.5152	676.5151	− 0.1	16:4–16:0	C_40_H_70_O_7_N
DGTS(30:5)	674.4996	674.5002	0.9	16:4–14:1	C_40_H_68_O_7_N
DGTS(32:0)	712.6091	712.6067	− 3.4	16:0–16:0	C_42_H_82_O_7_N
DGTS(32:1)	710.5935	710.5933	− 0.3	16:1–16:0	C_42_H_80_O_7_N
DGTS(32:2)	708.5778	708.5772	− 0.8	16:0–16:2 and 16:1–16:1	C_42_H_78_O_7_N
DGTS(32:3)	706.5622	706.5610	− 1.7	16:3–16:0	C_42_H_76_O_7_N
DGTS(32:4)	704.5465	704.5464	− 0.1	14:0–18:4, 16:4–16:0 and 16:3–16:1	C_42_H_74_O_7_N
DGTS(32:5)	702.5309	702.5306	− 0.4	**	C_42_H_72_O_7_N
DGTS(32:6)	700.5152	700.5152	0.0	16:4–16:2 and 16:3–16:3	C_42_H_70_O_7_N
DGTS(32:7)	698.4996	698.4993	− 0.4	16:4–16:3	C_42_H_68_O_7_N
DGTS(32:8)	696.4839	696.4844	0.7	16:4–16:4	C_42_H_66_O_7_N
DGTS(34:1)	738.6248	738.6251	0.4	16:0–18:1	C_44_H_84_O_7_N
DGTS(34:2)	736.6091	736.6056	− 4.8	16:1–18:1, 16:0–18:2 and 20:2–14:0	C_44_H_82_O_7_N
DGTS(34:3)	734.5935	734.5929	− 0.8	16:2–18:1, 16:1–18:2 and 20:3–14:0	C_44_H_80_O_7_N
DGTS(34:4)	732.5778	732.5776	− 0.3	18:4–16:0 and 18:3–16:1	C_44_H_78_O_7_N
DGTS(34:5)	730.5622	730.5616	− 0.8	18:4–16:1, 18:1–16:4 and 18:2–16:3	C_44_H_76_O_7_N
DGTS(34:5-OH)	746.5571	746.5575	0.5	16:4-OH-18:1, 16:1-OH-18:4 and 16:0-OH-18:5	C_44_H_76_O_8_N
DGTS(34:6)	728.5465	728.5458	− 1.0	**	C_44_H_74_O_7_N
DGTS(34:7)	726.5309	726.5306	− 0.4	16:4–18:3 and 16:3–18:4	C_44_H_72_O_7_N
DGTS(34:7-OH)	742.5258	742.5258	0.0	16:4-OH-18:3 and 16:3-OH-18:4	C_44_H_72_O_8_N
DGTS(34:8)	724.5152	724.5149	− 0.5	16:4–18:4	C_44_H_70_O_7_N
DGTS(36:10)	748.5152	748.5140	− 1.6	18:5–18:5	C_46_H_70_O_7_N
DGTS(36:4)	760.6091	760.6098	0.9	16:0–20:4, 18:3–18:1, 18:2–18:2, 20:3–16:1, 20:2–16:2, 20:1–16:3 and 20:0–16:4	C_46_H_82_O_7_N
DGTS(36:5)	758.5935	758.5933	− 0.3	18:4–18:1, 20:5–16:0, 20:4–16:1, 20:3–16:2, 20:2–16:3, 20:1–16:4 and 16:1–20:4	C_46_H_80_O_7_N
DGTS(36:6)	756.5778	756.5775	− 0.4	16:1–20:5, 20:3–16:3, 18:2–18:4 and 18:3–18:3	C_46_H_78_O_7_N
DGTS(36:7)	754.5622	754.5621	− 0.1	18:4–18:3	C_46_H_76_O_7_N
DGTS(36:8)	752.5465	752.5462	− 0.4	18:4–18:4	C_46_H_74_O_7_N
DGTS(36:9)	750.5309	750.5281	− 3.7	18:5–18.4	C_46_H_72_O_7_N
DGTS(38:10)	776.5465	776.5464	− 0.2	20:5–18:5	C_48_H_74_O_7_N
DGTS(38:7)	782.5935	782.5929	− 0.8	20:5–18:2	C_48_H_80_O_7_N
DGTS(38:8)	780.5778	780.5743	− 4.5	20:5–18:3	C_48_H_78_O_7_N
DGTS(40:10)	804.5778	804.5772	− 0.7	20:5–20:5	C_50_H_78_O_7_N
DGTS(40:9)	806.5935	806.5905	− 3.7	20:4–20:5	C_50_H_80_O_7_N

*C* number of carbon atoms, *N* number of double bonds.

*Identified based on the polar head fragmentation, calculated mass, and retention time.

**Identified according to the calculated mass and the retention time.

The most abundant ions in each class of betaine lipids were MGTS (18:4) and DGTS (34:4) assigned as DGTS (18:4–16:0) and DGTS (18:3–16:1) (Table 4). Interestingly, among the identified DGTS molecular ions, oxidized DGTS molecular ions were also found, the most abundant being DGTS (34:7-OH), identified as a combination of two molecular species of DGTS (16:4-OH-18:3) and DGTS (16:3-OH-18:4). These oxidized ions were identified based on exact mass measurements, retention times, and by analysis of MS/MS spectra (Supplementary Table S3). The MS/MS of the oxidized DGTS molecular ions showed a neutral loss of H_2_O and the product ions formed due to the loss of oxidized fatty acyl chains (acid and keto derivatives). Also, the product ion characteristic of betaine lipids at *m/z* 236 was present in all MS/MS spectra, therefore no product ions with oxidized polar head groups were observed.

**Figure 4:**
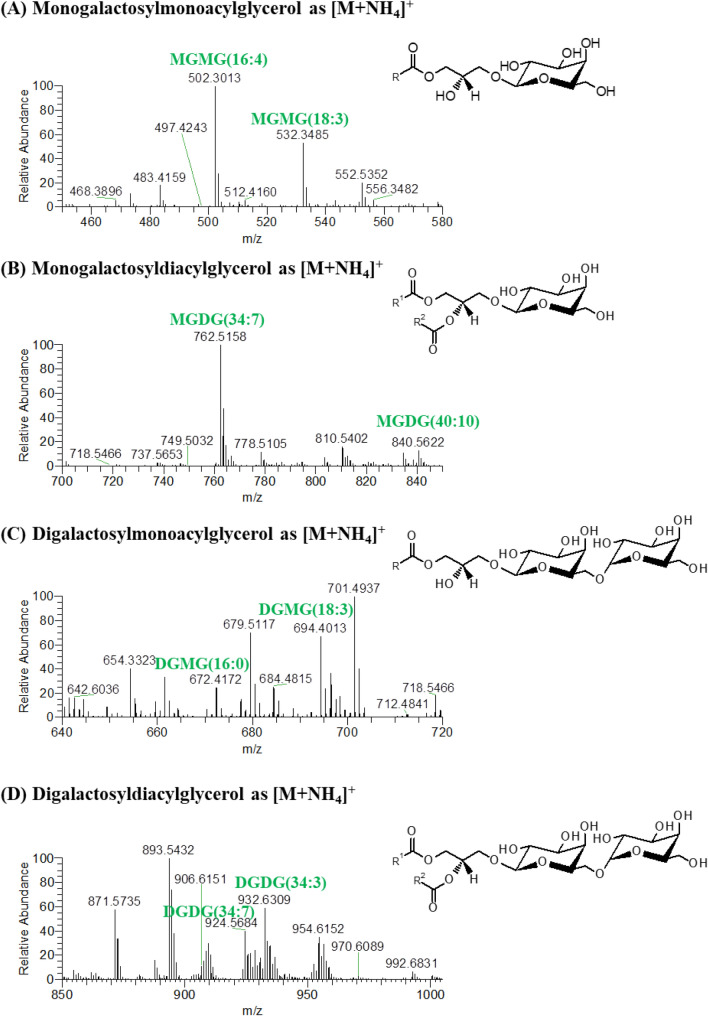
HILIC–HR–ESI–MS spectra of the classes of glycolipids (GL) identified in positive ion mode as [M+NH_4_]^+^. These classes were (**A**) monogalactosylmonoacylglycerol (MGMG), (**B**) monogalactosyldiacylglycerol (MGDG), (**C**) digalactosylmonoacylglycerol (DGMG) and (**D**) digalactosyldiacylglycerol (DGDG).

**Figure 5:**
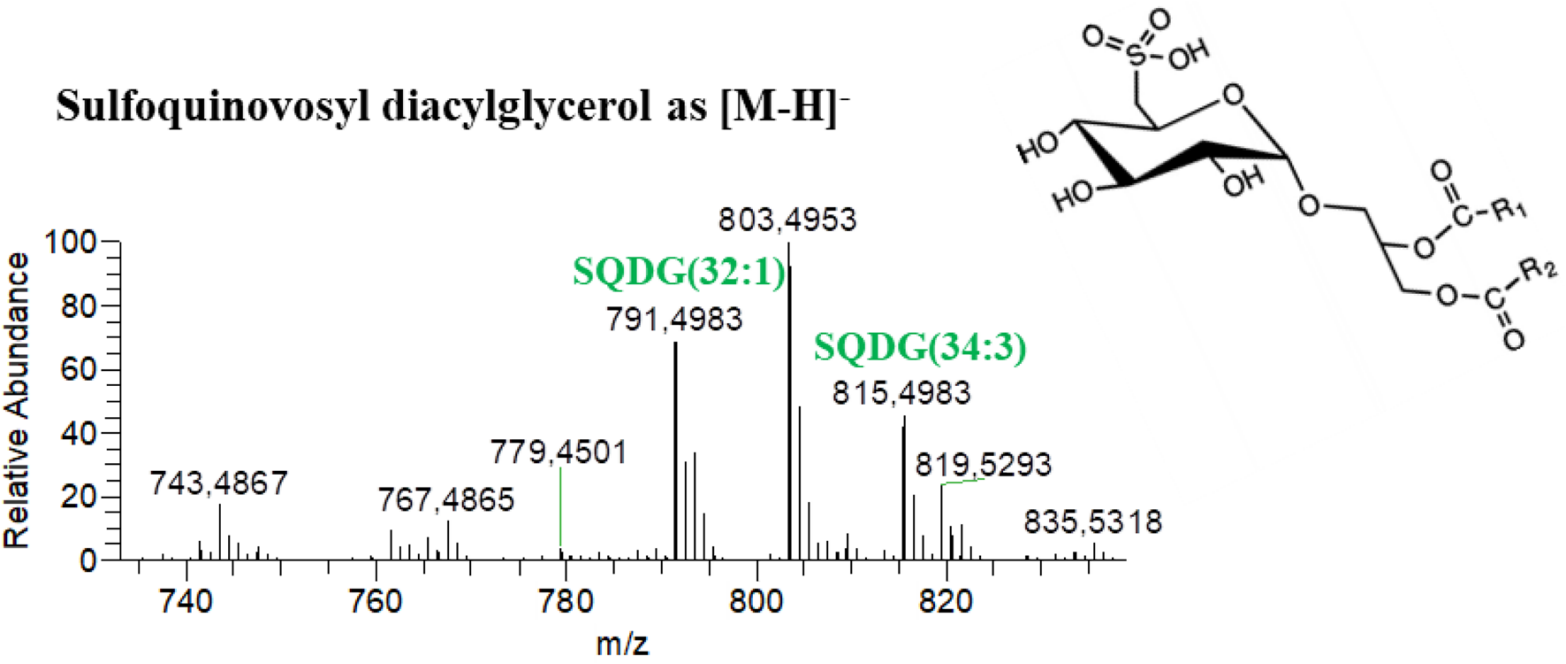
HILIC–HR–ESI–MS spectrum of the classes of glycolipids (GL) identified in negative ion mode as [M−H]^−^. This class was sulfoquinovosyl diacylglycerol (SQDG).

The glycolipids identified in the *Chlorococcum amblystomatis* samples included monogalactosyldiacylglycerol (MGDG), monogalactosylmonoacylglycerol (MGMG), digalactosyldiacylglycerol (DGDG) and digalactosylmonoacylglycerol (DGMG), identified as [M+NH_4_]^+^ ions (Fig. 4, Table 5). We also identified sulfoquinovosyl diacylglycerol lipids (SQDG) and a sulfoquinovosyl monoacylglycerol lipid (SQMG), identified as [M−H]^−^ ions (Fig. 5, Table 5). Analysis of the HILIC–HR–ESI–MS and MS/MS spectra identified the following number of molecular ions: 30 MGDG, 11 MGMG, 27 DGDG, 11 DGMG, 21 SQDG and 1 SQMG (Supplementary Table S2). From these molecular ions, we identified the following number of molecular species using MS/MS data: 47 MGDG, 11 MGMG, 48 DGDG, 11 DGMG, 40 SQDG and 1 SQMG.

**Table 5:** Glycolipids identified in the polar lipidome of *Chlorococcum amblystomatis* by HILIC–HR–ESI–MS and MS/MS**.**

Lipid species (C:N)	Calculated *m/z*	Observed *m/z*	Error (ppm)	Fatty acyl chains (C:N)	Formula
**MGMG identified as [M+NH**_**4**_**]**^**+**^					
MGMG(14:0)	482.3329	482.3324	− 1.1	14:0	C_23_H_48_NO_9_
MGMG(16:0)	510.3642	510.3639	− 0.6	16:0	C_25_H_52_NO_9_
MGMG(16:1)	508.3486	508.3482	− 0.7	16:1	C_25_H_50_NO_9_
MGMG(16:3)	504.3173	504.3174	0.3	**	C_25_H_46_NO_9_
MGMG(16:4)	502.3016	502.3013	− 0.6	16:4	C_25_H_44_NO_9_
MGMG(16:4-OH)	518.2965	518.2963	− 0.4	16:4-OH	C_25_H_44_NO_9_O
MGMG(18:1)	536.3799	536.3793	− 1.0	18:1	C_27_H_54_NO_9_
MGMG(18:3)	532.3486	532.3485	− 0.1	18:3	C_27_H_50_NO_9_
MGMG(18:4)	530.3329	530.3330	0.2	*	C_27_H_48_NO_9_
MGMG(20:4)	558.3642	558.3636	− 1.1	**	C_29_H_52_NO_9_
MGMG(20:5)	556.3486	556.3482	− 0.6	**	C_29_H_50_NO_9_
**MGDG identified as [M+NH**_**4**_**]**^**+**^					
MGDG(30:0)	720.5625	720.5593	− 4.4	**	C_39_H_78_NO_10_
MGDG(30:1)	718.5464	718.5466	0.3	16:1–14:0	C_39_H_76_NO_10_
MGDG(32:0)	748.5939	748.5910	− 3.8	16:0–16:0	C_41_H_82_NO_10_
MGDG(32:1)	746.5777	746.5780	0.4	16:1–16:0	C_41_H_80_NO_10_
MGDG(32:2)	744.5626	744.5624	− 0.3	16:1–16:1, 14:0–18:2 and 16:0–16:2	C_41_H_78_NO_10_
MGDG(32:3)	742.5464	742.5467	0.4	**	C_41_H_76_NO_10_
MGDG(32:4)	740.5307	740.5305	− 0.3	**	C_41_H_74_NO_10_
MGDG(32:5)	738.5156	738.5158	0.2	**	C_41_H_72_NO_10_
MGDG(32:6)	736.4994	736.4999	0.7	**	C_41_H_70_NO_10_
MGDG(32:7)	734.4843	734.4835	− 1.1	**	C_41_H_68_NO_10_
MGDG(32:8)	732.4687	732.4686	− 0.1	16:4–16:4	C_41_H_66_NO_10_
MGDG(34:1)	774.6090	774.6073	− 2.2	**	C_43_H_84_NO_10_
MGDG(34:2)	772.5933	772.5926	− 0.9	18:2–16:0	C_43_H_82_NO_10_
MGDG(34:3)	770.5782	770.5782	0.0	18:3–16:0 and 18:2–16:1	C_43_H_80_NO_10_
MGDG(34:4)	768.5626	768.5645	2.5	16:2–18:2, 16:1–18:3, 16:0–18:4, 16:3–18:1 and 16:4–18:0	C_43_H_78_NO_10_
MGDG(34:5)	766.5469	766.5474	0.6	*	C_43_H_76_NO_10_
MGDG(34:7)	762.5156	762.5158	0.2	16:4–18:3 and 18:4–16:3	C_43_H_72_NO_10_
MGDG(34:8)	760.5000	760.4999	− 0.1	**	C_43_H_70_NO_10_
MGDG(36:3)	798.6095	798.6055	− 5.0	**	C_45_H_84_NO_10_
MGDG(36:4)	796.5933	796.5892	− 5.1	**	C_45_H_82_NO_10_
MGDG(36:5)	794.5782	794.5778	− 0.5	18:3–18:2, 18:1–18:4, 20:5–16:0, 16:4–20:2 and 16:1–20:4	C_45_H_80_NO_10_
MGDG(36:6)	792.5625	792.5623	− 0.3	18:3–18:3, 16:1–20:5 and 16:4–20:2	C_45_H_78_NO_10_
MGDG(36:7)	790.5469	790.5470	0.1	**	C_45_H_76_NO_10_
MGDG(36:8)	788.5313	788.5339	3.3	**	C_45_H_74_NO_10_
MGDG(38:6)	820.5939	820.5931	− 1.0	18:1–20:5, 18:2–20:4 and 18:3–20:3	C_47_H_82_NO_10_
MGDG(38:7)	818.5782	818.5777	− 0.6	20:5–18:2 and 18:3–20:4	C_47_H_80_NO_10_
MGDG(38:8)	816.5626	816.5614	− 1.4	**	C_47_H_78_NO_10_
MGDG(40:10)	840.5626	840.5622	− 0.4	20:5–20:5	C_49_H_78_NO_10_
MGDG(40:8)	844.5939	844.5916	− 2.7	20:4–20:4	C_49_H_82_NO_10_
MGDG(40:7)	846.6095	846.6097	0.2	**	C_49_H_84_NO_10_
**DGMG identified as [M+NH**_**4**_**]**^**+**^					
DGMG(14:0)	644.3857	644.3848	− 1.4	**	C_29_H_58_NO_14_
DGMG(16:0)	672.4170	672.4172	0.3	16:0	C_31_H_62_NO_14_
DGMG(16:1)	670.4014	670.4012	− 0.3	16:1	C_31_H_60_NO_14_
DGMG(16:2)	668.3857	668.3854	− 0.5	**	C_31_H_58_NO_14_
DGMG(16:3)	666.3701	666.3699	− 0.3	**	C_31_H_56_NO_14_
DGMG(16:4)	664.3544	664.3541	− 0.5	**	C_31_H_54_NO_14_
DGMG(18:1)	698.4327	698.4327	0.0	18:1	C_33_H_64_NO_14_
DGMG(18:2)	696.4170	696.4149	− 3.0	**	C_33_H_62_NO_14_
DGMG(18:3)	694.4014	694.4013	− 0.1	18:3	C_33_H_60_NO_14_
DGMG(18:4)	692.3857	692.3856	− 0.2	**	C_33_H_58_NO_14_
DGMG(20:5)	718.4014	718.4009	− 0.7	**	C_35_H_60_NO_14_
**DGDG identified as [M+NH**_**4**_**]**^**+**^					
DGDG(30:0)	882.6154	882.6111	− 4.9	**	C_45_H_88_O_15_N
DGDG(30:1)	880.5997	880.5990	− 0.8	16:1–14:0	C_45_H_86_O_15_N
DGDG(32:1)	908.6310	908.6306	− 0.4	16:0–16:1	C_47_H_90_O_15_N
DGDG(32:2)	906.6154	906.6151	− 0.3	16:0–16:2 and 16:1–16:1	C_47_H_88_O_15_N
DGDG(32:3)	904.5997	904.6011	1.5	18:3–14:0, 16:0–16:3 and 16:1–16:2	C_47_H_86_O_15_N
DGDG(32:4)	902.5841	902.5846	0.6	18:3–14:1, 16:0–16:4 and 14:0–18:4	C_47_H_84_O_15_N
DGDG(32:5)	900.5684	900.5678	− 0.7	16:1–16:4	C_47_H_82_O_15_N
DGDG(32:6)	898.5528	898.5512	− 1.8	**	C_47_H_80_O_15_N
DGDG(34:1)	936.6623	936.6615	− 0.9	18:1–16:0	C_49_H_94_O_15_N
DGDG(34:2)	934.6467	934.6443	− 2.6	18:1–16:1 and 18:2–16:0	C_49_H_92_O_15_N
DGDG(34:3)	932.6310	932.6309	− 0.1	16:0–18:3 and 18:1–16:2	C_49_H_90_O_15_N
DGDG(34:4)	930.6154	930.6143	− 1.2	18:1–16:3, 18:2–16:2, 18:3–16:1, 18:4–16:0 and 14:0–20:4	C_49_H_88_O_15_N
DGDG(34:5)	928.5997	928.5991	− 0.6	18:3–16:2, 18:2–16:3, 18:1–16:4, 16:1–18:4 and 14:0–20:5	C_49_H_86_O_15_N
DGDG(34:6)	926.5841	926.5820	− 2.3	**	C_49_H_84_O_15_N
DGDG(34:7)	924.5684	924.5684	0.0	18:3–16:4 and 18:4–16:3	C_49_H_82_O_15_N
DGDG(34:8)	922.5528	922.5501	− 2.9	**	C_49_H_80_O_15_N
DGDG(36:2)	962.6780	962.6751	− 3.0	**	C_51_H_96_O_15_N
DGDG(36:3)	960.6623	960.6606	− 1.8	18:3–18:0 and 18:1–18:2	C_51_H_94_O_15_N
DGDG(36:4)	958.6467	958.6433	− 3.5	**	C_51_H_92_O_15_N
DGDG(36:5)	956.6310	956.6301	− 0.9	20:5–16:0, 18:1–18:4 and 20:1–16:4	C_51_H_90_O_15_N
DGDG(36:6)	954.6154	954.6152	− 0.2	18:3–18:3, 20:5–16:1 and 18:4–18:2	C_51_H_88_O_15_N
DGDG(36:7)	952.5997	952.5999	0.2	18:3–18:4	C_51_H_86_O_15_N
DGDG(36:8)	950.5841	950.5835	− 0.6	18:4–18:4	C_51_H_84_O_15_N
DGDG(38:6)	982.6467	982.6421	− 4.7	**	C_53_H_92_O_15_N
DGDG(38:7)	980.6310	980.6312	0.2	18:2–20:5	C_53_H_90_O_15_N
DGDG(40:10)	1002.6154	1002.6148	− 0.6	20:5–20:5	C_55_H_88_O_15_N
DGDG(40:9)	1004.6310	1004.6323	1.3	**	C_55_H_90_O_15_N
**SQDG identified as [M−H]**^**−**^					
SQDG(28:0)	737.4510	737.4492	− 2.4	**	C_37_H_69_O_12_S
SQDG(30:0)	765.4823	765.4818	− 0.6	14:0–16:0	C_39_H_73_O_12_S
SQDG(30:1)	763.4666	763.4667	0.1	14:0–16:1	C_39_H_71_O_12_S
SQDG(32:0)	793.5136	793.5135	− 0.1	**	C_41_H_77_O_12_S
SQDG(32:1)	791.4979	791.4982	0.3	16:0–16:1	C_41_H_75_O_12_S
SQDG(32:2)	789.4823	789.4821	− 0.2	16:1–16:1 and 16:0–16:2	C_41_H_73_O_12_S
SQDG(32:3)	787.4666	787.4668	0.2	16:1–16:2, 16:0–16:3 and 18:1–14:1	C_41_H_71_O_12_S
SQDG(32:4)	785.4510	785.4517	0.9	16:0–16:4, 16:1–16:3 and 16:2–16:2	C_41_H_69_O_12_S
SQDG(34:0)	821.5449	821.5446	− 0.3	16:0–18:0	C_43_H_81_O_12_S
SQDG(34:1)	819.5292	819.5296	0.5	16:0–18:1	C_43_H_79_O_12_S
SQDG(34:3)	815.4979	815.4982	0.3	16:0–18:3 and 16:2–18:1	C_43_H_75_O_12_S
SQDG(34:4)	813.4823	813.4829	0.8	16:0–18:4, 16:1–18:3, 16:2–18:2, 18:1–16:3 and 14:0–20:4	C_43_H_73_O_12_S
SQDG(34:5)	811.4666	811.4667	0.1	16:0–18:5, 16:1–18:4, 16:2–18:3, 16:3–18:2 and 16:4–18:1	C_43_H_71_O_12_S
SQDG(36:0)	849.5762	849.5761	− 0.1	16:0–20:0	C_45_H_85_O_12_S
SQDG(36:3)	843.5292	843.5287	− 0.6	**	C_45_H_79_O_12_S
SQDG(36:4)	841.5136	841.5135	− 0.1	16:0–20:4	C_45_H_77_O_12_S
SQDG(36:5)	839.4979	839.4987	0.9	**	C_45_H_75_O_12_S
SQDG(36:6)	837.4823	837.4823	0.0	16:1–20:5, 20:4–16:2, 18:2–18:4 and 18:3–18:3	C_45_H_73_O_12_S
SQDG(36:7)	835.4666	835.4676	1.2	18:3–18:4, 20:4–16:3 and 20:5–16:2	C_45_H_71_O_12_S
SQDG(34:3-OH)	831.4928	831.4930	0.2	–	C_43_H_75_O_13_S
SQDG(34:4-OH)	829.4772	829.4752	− 2.4	–	C_43_H_73_O_13_S
**SQMG identified as [M−H]**^**−**^					
SQMG(16:0)	555.2839	555.2838	− 0.2	16:0	C_25_H_47_O_11_S

*C* carbons, *N* number of double bonds.

*Identified based on the polar head fragment, calculated mass and retention time.

**Identified based on calculated mass and retention time.

The typical fragmentation observed in the HILIC–HR–ESI–MS/MS spectra of neutral glycolipids, as [M+NH_4_]^+^ ions, allowed to confirm the polar head group by the neutral loss of 197 Da (for MGDG) or 359 Da (for DGDG), which corresponds to the loss of the carbohydrate moiety combined with the loss of NH_3_^44,47^. The assignment of the fatty acyl composition was corroborated by the presence of product ions corresponding to each fatty acyl group as acylium plus 74 [RCO+74]^+^ ion^44,47^ (Supplementary Table S2). On the other hand, the HILIC–HR–ESI–MS/MS spectra of the [M−H]^−^ ions of the acidic glycolipids (SQDG and SQMG), when available, showed the product ion of the sulfoquinovosyl polar head group at *m/z* 225 and the fatty acyl composition was confirmed by the neutral loss of fatty acyl chains as carboxylic acid (-RCOOH) and by the presence of carboxylate RCOO^−^ anions (Supplementary Table S2).

The most abundant species in each class of glycolipids were: MGDG (34:7), identified as MGDG (16:4–18:3) and MGDG (18:4–16:3); MGMG (16:4); DGDG (34:3), identified as DGDG (16:0–18:3) and DGDG (18:1–16:2); DGMG (18:3); and SQDG (32:1), identified as SQDG (16:0–16:1). The only identified glycolipid species belonging to the SQMG class was identified by accurate mass measurements and by the presence on the MS/MS spectrum of the product ion corresponding to the polar head at *m/z* 225. Among the MGMG and SQDG species identified, we observed three oxidized species, MGMG (16:4-OH), SQDG (34:3-OH) identified as SQDG (18:3-OH-16:0) and SQDG (34:4-OH) identified as SQDG (18:4-OH-16:0). HILIC–HR–ESI–MS/MS analysis of all oxidized GL species suggested that a hydroxyl group was present in the fatty acyl group (Supplementary Table S3).

**Figure 6:**
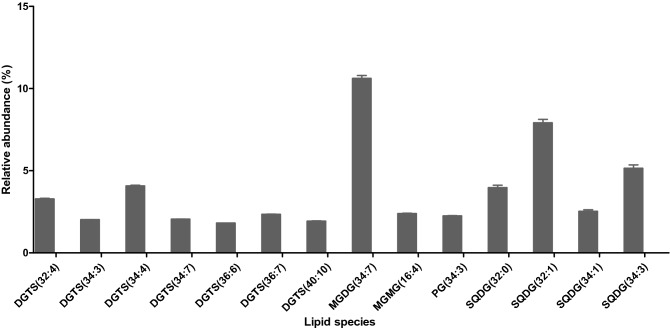
The most abundant lipid species, identified as molecular ions, in the polar lipidome of *Chlorococcum amblystomatis*, with a relative amount ≥ 1.5%. The results are represented by an average of five samples ± standard deviations.

The relative quantification of the identified species was carried out as described in the methods section. Figure 6 shows the most abundant lipid species, identified as molecular ions, in the polar lipidome of *Chlorococcum amblystomatis*, with a relative amount greater than or equal to 1.5%. Glycolipids were the group of lipids with the largest number of species (101). The most abundant species was MGDG (34:7), assigned as MGDG (16:3–18:4) and MGDG (16:4–18:3), and the second most abundant species was SQDG (16:0–16:1).

**Figure 7:**
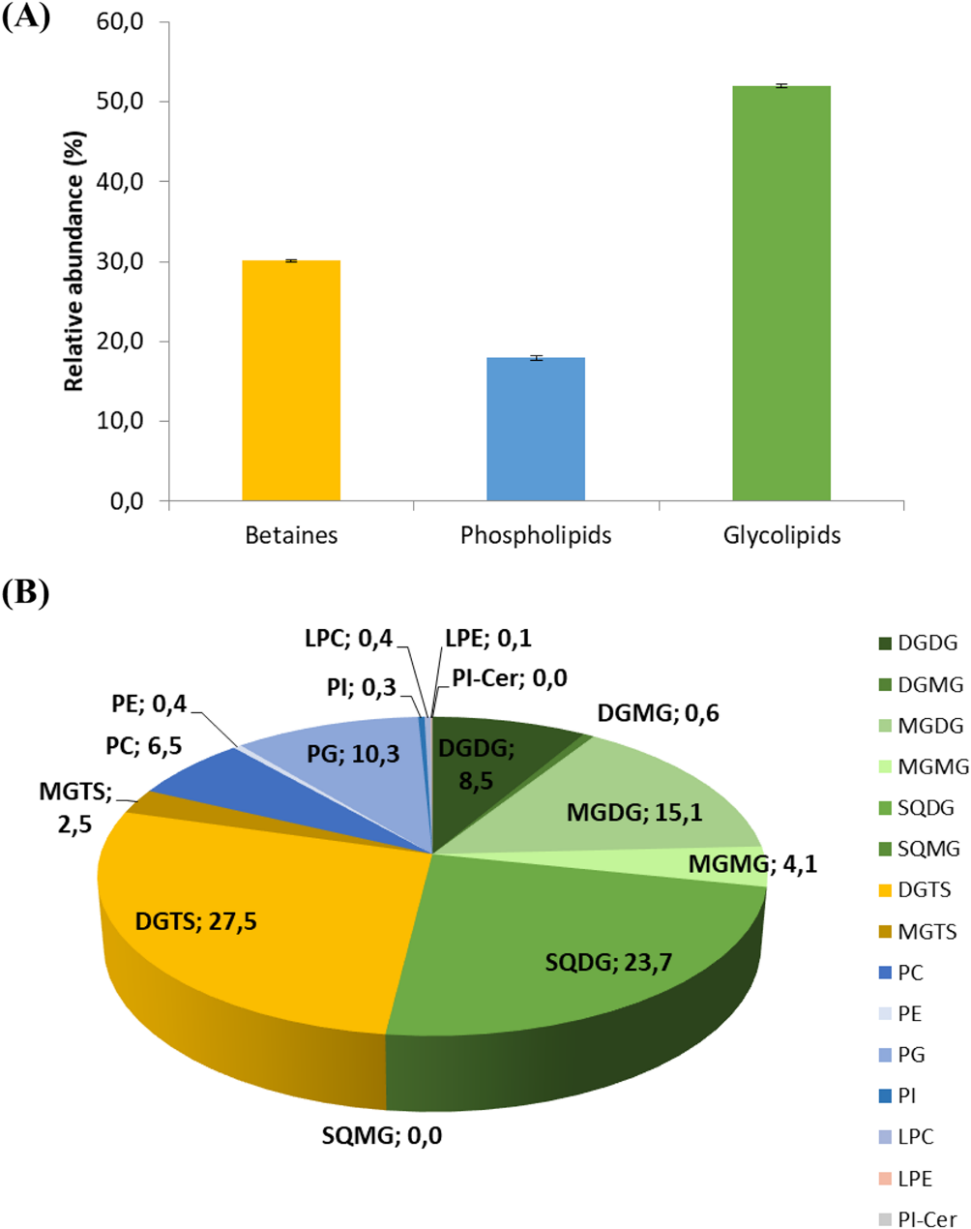
(**A**) Representation of the relative percentage of betaines, phospholipids and glycolipids present in *Chlorococcum amblystomatis*. Values are represented by an average of five replicates ± standard deviation. (**B**) The relative percentage of each class of lipids.

LC–MS data was acquired using an internal standard for each class of phospholipids. LC–MS data were normalized against their assigned internal standard, to semi-quantify each molecular ion. Due to the lack of commercially available GL and BL standards, internal standards from classes with a retention time closer to those identified for GL and BL were used to normalize their respective data, as performed in our laboratory^45,52–54^. The use of one internal standard is acceptable for semi-quantification because the ionization efficiency of polar lipids depends mainly on the polar head, while the length of the chain and the degree of unsaturation contribute little^55,56^. However, we would like to point out the limitations of our inter-class quantitation approach, because despite the similar retention time, the response factors for each class, even between phospholipids, tend to differ in ESI–MS, which prevents a robust and accurate quantification^51–53^. Normalized data were then used to semi-quantify the abundances of the identified molecular ions. For a clearer overview of the composition of the polar lipidome of *Chlorococcum amblystomatis*, we have summed up the relative percentage of all the detected lipid species belonging to glycolipids, betaines and phospholipids (Fig. 7A). According to the largest number of molecular ions, the highest cumulative levels were observed for glycolipids (51.9%), followed by betaine lipids (30.1%) and phospholipids (17.9%). The sum of the relative percentage of all lipid species belonging to each class of lipids was also calculated. The highest cumulative levels were observed for DGTS (27.6%) followed by SQDG (23.7%) (Fig. 7B).

### Evaluation of in vitro anti-inflammatory and antioxidant properties of *Chlorococcum amblystomatis* lipid extracts

**Figure 8:**
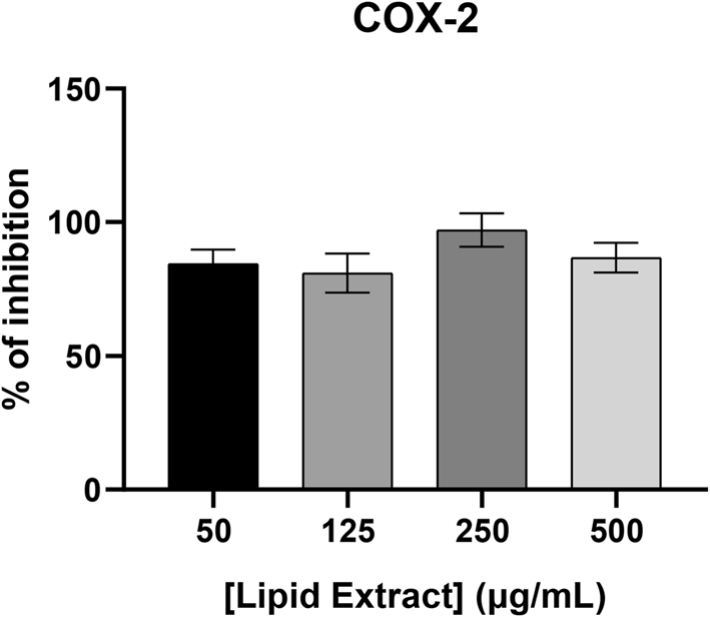
Percent inhibition of COx-2 as a function of the concentration of the lipid extracts of *Chlorococcum amblystomatis.*

The anti-inflammatory potential of lipid extracts from *Chlorococcum amblystomatis* were evaluated using a test kit for screening for inhibition of human COX-2. With 10 µl of an extract of 50 µg mL^−1^ (5 µg), we have observed an inhibition of 87.5 ± 0.1 of COX-2 activity (Fig. 8). These results suggest that the lipid extracts of *Chlorococcum amblystomatis* have anti-inflammatory potential.

**Figure 9:**
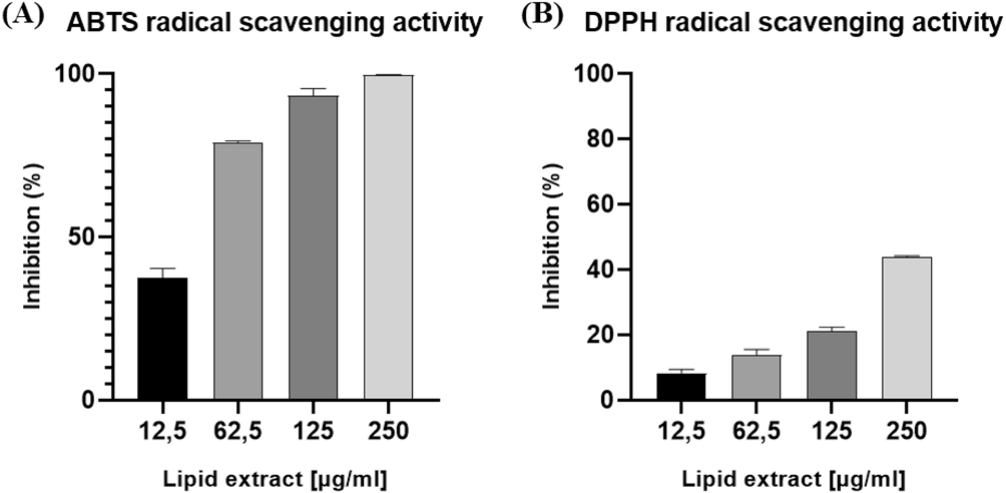
Percent inhibition as a function of the concentration of the lipid extracts of *Chlorococcum amblystomatis* after 120 min of ABTS^•+^ (**A**) and DPPH^•^ (**B**) radical scavenging activity.

The antioxidant potential of lipid extracts from *Chlorococcum amblystomatis* were evaluated using free radical DPPH^•^ and ABTS^•+^ scavenging assays (Fig. 9), as described in the methods section. The results obtained for the DPPH assay revealed that the concentration of the extract resulting in a 40% inhibition (IC40) of DPPH^•^ was at an estimated concentration of 226.81 ± 2.99 µg mL^−1^. The average antioxidant activity (Trolox equivalent, TE*)* was 76.25 ± 1.02 Trolox µmol g^−1^ of lipid extract. In contrast, the lipid extract which resulted in a 50% inhibition (IC50) of ABTS^•+^ was 37.69 ± 4.44 µg mL^−1^ and the expressed TE activity of 428.81 ± 54.32 Trolox µmol g^−1^ of lipid extract.

## Discussion

*Chlorococcum amblystomatis* is a green microalga with great potential to be a sustainable resource of commercially important essential lipids^1,2,29^, with promising applications in several industrial sectors^32,33^. In our work, the lipid extracts represented 20.77 ± 0.57%, of the dry weight of the biomass, which is consistent with the lipid content reported for *Chlorococcum* (8.71–32.3%)^10,11,18–20^. This wide range of lipid content determined by different studies is consistent with the variation in lipid content due to growing conditions^32,36,38–40^ and the use of different extraction methods^54^.

The FA profile identified by GC–MS included as the most abundant FA: C16:0, C18:3 (*n*-3), C18:0, C16:4 (*n*-3), C20:5 (*n*-3) and C16:1–9, representing respectively 23%, 19%, 14%, 11%, 9% and 7% of the total FA pool. It is important to note that this is the first time that FA C16:4 (*n*-3) and C20:5 (*n*-3) have been reported in *Chlorococcum amblystomatis*. The values of relative abundance collected in the present work are different from those reported in the literature to *Chlorococcum* sp^32,33,38,39^. Such differences could result from differences in growth conditions ^55^, different strains, different species, different derivatization methods or even GC detectors (FID or MS) ^56^. *Chlorococcum amblystomatis* extracts were rich in omega-3 PUFAs, representing 43.2%, with a high contribution of the most abundant FA, C18:3 (*n*-3), C16:4 (*n*-3) and C20:5 (*n*-3). Omega-3 PUFAs are known to have a variety of health benefits, such as preventing chronic diseases, as they are associated with anti-inflammatory and antioxidant protection, benefits for the cardiovascular system, prevention of breast cancer, improvement of neurological capacities and visual development^57–59^. To understand the beneficial impact on health and assess the nutritional quality of *Chlorococcum amblystomatis*, we calculated the values of AI (0.5), TI (0.3), h/H (1.4), and *n*-6/*n*-3 (0.1). The AI and TI indexes are commonly used to assess the potential of the matrix to stimulate platelet aggregation, and the lower the indexes the more beneficial it is in reducing the prevalence of heart disease ^60^, and have already been used to assess these benefits in microalgae, seaweeds and fish^61–65^. Our results show that these indexes of the lipid extract of *Chlorococcum amblystomatis* were similar to those observed in fish oils^66,67^. Compared to *Spirulina platensis*, *Nannochloropsis gaditana*,* Nannochloropsis oculata* and *Porphyridium tricornutum*, *Chlorococcum amblystomatis* had lower AI values. Their AI values ranged between 0.6 and 1.7 ^61^*.* As for the TI index, *Chlorococcum amblystomatis* had lower values than *Spirulina platensis*, *Nannochloropsis gaditana* and *Porphyridium Cruentum* ^61^, which were 1.5, 3.8 and 0.6, respectively. The higher values for h/H and the lower *n*-6/*n*-3 ratio (1.4 and 0.1, respectively) reinforce the nutritional potential of the lipid extracts of *Chlorococcum amblystomatis*.

The profiling of the total lipid extract using high-resolution HILIC–ESI–MS and MS–MS approaches, made it possible, for the first time, to describe the polar lipid composition, in *Chlorococcum amblystomatis*. The polar lipids identified included glycolipids (GL), phospholipids (PL) and betaine lipids (BL). Data from the literature indicate that algae are a valuable and promising source of polar lipids with bioactive properties, namely, polar lipids esterified to commercially important PUFAs, e.g. eicosapentaenoic acid (EPA). Our profiling made it possible to identify 245 molecular ions, corresponding to a minimum of 350 molecular species: 89 molecular ions of PL, 101 molecular ions of GL (sulfo- and galactolipids), 54 molecular ions of BL and 1 inositol phosphoceramide. These ions were distributed by 15 classes of lipids: PL classes were LPC, PC, LPE, PE, PG and PI; sphingolipid classes were PI-Cer, BL classes were MGTS and DGTS; and GL classes were MGMG, MGDG, DGMG, DGDG, SQDG, and SQDG.

About 45% of the lipid species detected were esterified to omega-3 fatty acids. Some of these species caught our attention because of their richness in omega-3 PUFAs. The PG (34:3) and the PG (36:5), respectively assigned to PG (16:0–18:3) and PG (16:0–20:5) are two species of PL among the most abundant of *Chlorococcum amblystomatis*, and both carried omega-3 fatty acids, including EPA. In a previous study in which anti-inflammatory activity was addressed, PG (16:0–20:5) was suggested to promote down-regulated iNOS activity in LPS-stimulated macrophages^21,68^.

Since in *Chlorococcum amblystomatis* there are high amounts of PLs esterified to omega-3 PUFAs and it has been suggested that the PLs are an excellent delivery vehicle for PUFAs^18,19^, this will favour its valorization for food and nutraceutical formulations, where they can be used as value-added ingredients, and also in the cosmetic industry, as they can be used to create moisturizing emulsions ^69^. Interestingly, oxidized phosphatidylglycerols (PG) with bonded oxylipins have been identified in this study. Some studies have suggested the anti-inflammatory potential of oxylipins ^70^, as reported for *Nannochloropsis gaditana* and *Chlamydomonas debaryana* ^71^. Indeed, we observed a COX-2 inhibitory activity from the lipid extracts of *Chlorcococcum amblystomatis*, to which these oxylipins could have contributed. Consequently, extracts rich in polar lipids from *Chlorococcum amblystomatis* could constitute an interesting opportunity for the nutraceutical or pharmaceutical industries.

The BL identified in the polar lipidome of *Chlorococcum amblystomatis*, include as the most abundant species, DGTS (34:4), DGTS (32:4), DGTS (36:7), DGTS (34:7), DGTS (34:3), DGTS (40:10) and DGTS (36:6). Moreover, DGTS (40:10) was identified as DGTS (20:5–20:5), a molecular species associated with anti-inflammatory activity, by reducing the production of nitric oxide (NO) ^72^. However, BL remains poorly studied to date, and little is known about its bioactive potential.

The polar lipidome of *Chlorococcum amblystomatis* was particularly rich in glycolipids (MGDG, DGDG and SQDG) which represented approximately half of the polar lipids content (Fig. 7). There is an interest in characterizing GL because of their reported bioactive properties ^16^. For example, SQDG of the microalga *Porphyridium purpureum* have been associated with antioxidant activity, due to their inhibitory effect on the generation of superoxide in peritoneal mononuclear cells ^73^. GL with PUFAs have shown anti-inflammatory activity by inhibiting the release of nitric oxide by macrophages^20,21^. There is also a patent for MGDG with EPA to be used as an anti-inflammatory compound ^74^. Another interesting application for glycolipids comes from the use of DGDG and SQDG from seaweeds as chemotherapy agents ^22^. SQDG esterified in EPA appears to have an anti-proliferative effect by inhibiting the key enzymes telomerase ^23^ and DNA polymerase-α and -β ^75^. GL species with known bioactivity, such as MGDG (20:5–16:0), MGDG (20:5–16:1), MGDG (20:5–18:2), MGDG (20:5–20:5), DGDG (20:5–16:0), DGDG (20:5–18:2), DGDG (18:4–16:0) and MGDG (16:4–18:3), were also identified in the *Chlorococcum amblystomatis* lipidome. These GL species were associated with anti-inflammatory activity by down-regulating the iNOS^46,76–78^.

Regarding sulfur-containing lipids, SQDG (34:3), assigned as SQDG (16:0–18:3), and SQDG (32:0), were among the most abundant SQDG identified in *Chlorococcum amblystomatis*, and were linked to anti-inflammatory ^79^ and antiviral activities ^80^, respectively. Also, SQDG (16:0–20:4) and SQDG (16:0–18:4), identified in extracts of *Chlorococcum amblystomatis*, have been reported for their antiproliferative properties^81,82^. Finally, SQMG (16:0), although detected in low abundance, has been reported to have antimicrobial and antitumor activities ^83^.

The polar lipids of *Chlorococcum amblystomatis* may contribute to the antioxidant activity observed in the DPPH and ABTS radical scavenging assays, as some of the reported polar lipids were previously associated with antioxidant activity ^73^. Natural antioxidants are highly sought after for their biological effects ^84^ and their wide applications in the food and pharmaceutical sectors ^85^. As such, microalgal biomass extracts have been widely explored regarding their potential antioxidant activity^86,87^. Ethanolic and aqueous extracts are mainly composed of phenolic compounds, while methanolic extracts are rich in polar lipids ^88^. In a recent study, the DPPH assay was employed to evaluate antioxidant activity on 9 microalgae. The methanolic extracts of the different microalgae strains showed antioxidant activity, with percentage inhibition of DPPH ranging from 15 to 45% (IC15 to IC45; at 200 μg mL^−1^ extracts concentration) ^86^. In this work, the dichloromethane:methanol extracts of *Chlorococcum amblystomatis* resulted in a percentage of inhibition of 36% at 200 µg mL^−1^, displaying a value better compared to 7 strains of microalgae from the reported work.

COX-2 is an important component of inflammation, associated with pro-inflammatory activity, responsible for the production of prostaglandin E_2_^89^. In the present work, the lipid extracts of *Chlorococcum amblystomatis* exhibit a COX-2 inhibiting activity. It is the first time that such a response has been described in *Chlorococcum* sp. However, recent work, also measuring COX-2 inhibition, compared the anti-inflammatory potential of aqueous and ethanolic extracts of two *Tetraselmis* sp. strains and a *Skeletonema* sp. strain ^81^. The highest value of anti-inflammatory activity (82 ± 2%) was measured for the ethanolic extract of *Skeletonema* sp. at a concentration of 1 mg mL^-1^. Our results showed that the COX inhibition of dichloromethane: methanol extracts of *Chlorococcum amblystomatis* was 87.5 ± 0.1%, at a concentration of 50 μg mL^−1^. These results are consistent with the hypothesis that *Chlorococcum amblystomatis* has a higher COX-2 inhibitory power than Tetraselmis sp. and Skeletonema sp. The high COX-2 inhibitory activity of Chlorococcum amblystomatis extracts results in promising anti-inflammatory potential. Future studies should explore the use of lipid extracts of Chlorococcum amblystomatis in inflammatory cells to explore this anti-inflammatory potential.

## Conclusions

The polar lipidome of *Chlorococcum amblystomatis* was characterized for the first time in this study. The *Chlorococcum amblystomatis* strain used revealed a high content of omega-3 PUFAs. PUFAs are associated with several health benefits, such as the prevention of cardiovascular disease. The HILIC–MS/MS lipidomic approach identified 245 molecular ions of polar lipids, in *Chlorococcum amblystomatis,* revealing to be a microalga rich in glyco- and betaine lipids. Some of the identified polar lipids have already been reported with biological activity, for example, DGTS (20:5–20:5), SQDG (16:0–18:3), MGDG (20:5–18:2) and DGDG (20:5–18:2) which were associated with anti-inflammatory activity. In addition, extracts rich in polar lipids had COX-2 inhibiting activity and antioxidant activity. In conclusion, due to its chemical, biochemical, bioactive, and health-promoting properties, the lipid extracts of *Chlorococcum amblystomatis* have been found to be of high value for application in food, feed, cosmetic, nutraceutical, and pharmaceutical applications.

## Materials and methods

### Reagents

HPLC grade methanol (MeOH), ethanol absolute, and dichloromethane (CH_2_Cl_2_), were purchased from Fisher Scientific Ltd. (Loughborough, UK). All other reagents were purchased from major commercial sources. Milli-Q water (Synergy, Millipore Corporation, Billerica, MA, USA) was used. Phospholipid internal standards 1,2-dimyristoyl-*sn*-glycero-3-phosphocholine (dMPC), 1,2-dimyristoyl-*sn*-glycero-3-phosphoethanolamine (dMPE), 1,2-dimyristoyl-*sn*-glycero-3-phospho-(10-rac-glycerol) (dMPG), 1,2-dimyristoyl-*sn*-glycero-3-phospho-l-serine (dMPS), 1,2-dipalmitoyl-*sn*-glycero-3-phosphatidylinositol (dPPI), N-palmitoyl-D-*erythro*-sphingosylphosphorylcholine (SM), 1-nonadecanoyl-2-hydroxy-*sn*-glycero-3-phosphocholine (LPC) were purchased from Avanti Polar Lipids, Inc. (Alabaster, AL), according to the methodology previously described^34,35^. DPPH^•^ was purchased from Aldrich (Milwaukee, WI). 2,20-Azino-bis(3-ethylbenzothiazoline-6-sulfonic acid) diammonium salt (ABTS^•+^) was obtained from Fluka (Buchs, Switzerland). The 6-hydroxy-2,5,7,8-tetramethylchromane-2-carboxylic acid (Trolox) were purchased from Sigma-Aldrich (St Louis, MO, USA). The cyclooxygenase (COX-2) inhibitory screening assay was performed using a commercial kit, Cayman test kit-701080 from Cayman Chemical Company (Ann Arbor, MI, USA). All the other reagents and chemicals used were of the highest grade of purity commercially available.

### Microalgae material

The spray-dried biomass of *Chlorococcum amblystomatis* was supplied by Allmicroalgae, Natural products S.A. located in Pataias, Portugal, Fábrica Cibra Pataias, 2445-287 Pataias. The strain was isolated locally and is deposited in the Allmicroalgae culture collection under the code 0066CA ^34^.

Portugal *Chlorococcum amblystomatis* 0066 CA was cultivated autotrophically in Guillard’s F2 culture medium, whose composition was adapted to local water, using nitrates as the source of nitrogen ^91^. Briefly, 5 L flask reactors were cultivated from 7 to 15 days, under continuous exposure to light. Five 5 L flask reactors were used to inoculate one 0.1 m^3^ L outdoor Flat Panel (FP) reactor, which was later sequentially scaled as follows: 0.25 m^3^ L to a 0.5 m^3^ L to a 1 m^3^ Flat Panels. Four of the later reactors were used as inoculum of a 10 m^3^ photobioreactor (PBR). The reactor was operated for 21 days, exposed to the environmental light and temperature conditions, at an average temperature of 15.5 °C and light irradiance of 20.10 MJ m^−2^ day^−1^. pH was maintained constant, 7.0–8.0, by pulse injections of CO_2_ and the temperature was kept under 28 °C by a sprinkler-like irrigation system. After growing period, the biomass was industrially collected by centrifugation and further spray-drying.

### Lipid extraction procedure

Lipid extraction was carried out using a mixture of dichloromethane: methanol solvents (DM) (2:1, v/v). The lipids were extracted from 25 mg of lyophilized *Chlorococcum amblystomatis* biomass using the solvent mixture. The suspension was centrifuged (Selecta JP Mixtasel, Abrera, Barcelona, Spain) at 2000 rpm for 10 min and the supernatant was collected in a new pre-weighed glass tube. This process was repeated four times until the extraction solvent lost the green colour. The combined supernatants were dried under a stream of nitrogen.

The Folch extraction method was used to the obtained dried supernatants^54,92^. The extracts were redissolved in 2 mL of dichloromethane, and 1 mL of methanol and 0.75 mL of Milli-Q water were added. The mixture was vortexed for 2 min followed by phase separation by centrifugation at 2000 rpm for 10 min. The organic phase was collected in a new pre-weighed tube, and the aqueous phase was reextracted with 2 mL of dichloromethane, two more times. The combined organic phases were dried under a stream of nitrogen and weighted.

Each series of extracts was repeated five times and the total lipid content was determined by gravimetry. The yield of lipids extracted from dry biomass extracts (DW) was calculated as follows (Eq. 1):

1 $${\text{Lipid content yield }}\left( {{\text{\% DW,}}\frac{{\text{w}}}{{\text{w}}}} \right){ = }\frac{{\text{Weight of the lipid extract (g)}}}{{\text{Weight of biomass (g)}}} \times {100}$$

### COX-2 inhibition assay

A commercial cyclooxygenase (COX-2) inhibitory screening assay kit—Cayman test kit-701080 (Cayman Chemical Company, Ann Arbor, MI, USA)—was used to assess their anti-inflammatory potential ^93^. This screening assay directly measures the amount of prostaglandin F2α generated from arachidonic acid (AA, C20:4 [*n*-6]) in the cyclooxygenase reaction. This assay was carried out according to the instructions described by the supplier of the assay kit, using an aliquot of test extract or DMSO. For this assay, lipid extracts of *Chlorococcum amblystomatis* (500 μg, 250 μg, 125 μg and 50 μg) were dissolved in DMSO and the final volume of reaction was 1000 μl. Positive and negative controls were provided by the assay kit protocol. The positive control used inactivated COX-2 enzyme, and negative control used the enzyme with 100% initial activity without any inhibitor. The assay was performed in three replicates. Interferences were considered by subtracting COX-2 inhibition from the blank assays. The results were expressed as a percentage of inhibited COX-2.

### DPPH Radical Scavenging Assay

The antioxidant scavenging activity against the α,α-diphenyl-β-picrylhydrazyl radical (DPPH^•^) was evaluated as described previously^94,95^ with some modifications. Briefly, 150 µL of an ethanolic dilution of the extracts (25, 125, 250, 500 µg mL^−1^) or 150 µL of the Trolox standard solution (5, 12.5, 25, 37.5 µmol L^−1^ in ethanol) were mixed in triplicate with 150 µL of a DPPH^•^ working solution in ethanol (absorbance ~ 0.9, 517 nm). The mixture was incubated for 120 min and the absorbance was measured at 517 nm every 5 min (Multiskan GO 1.00.38, Thermo Scientific, Hudson, NH, USA). A control was prepared by replacing the DPPH^•^ solution with ethanol. The antioxidant activity, expressed as a percentage of inhibition of the DPPH radical, was calculated using the following equation (Eq. 2):

2 $${\text{Inhibition\% }} = \frac{{\left( {Abs_{DPPH \bullet } - Abs_{Sample} } \right)}}{{Abs_{DPPH \bullet } }} \times 100$$

The activity is expressed in Trolox Equivalents, which were calculated using Eq. 2, where IC40 values are the concentration of sample or of Trolox that induces the reduction the DPPH• radical to 40%) (Eq. 3):

3 $${\text{TE}} = \frac{{{\text{IC}}40{\text{ Trolox }}\left( {\mu {\text{mol}}/{\text{g}}} \right)}}{{{\text{IC}}40{\text{ of samples }}\left( {\mu {\text{g}}/{\text{mL}}} \right)}} \times 1000.$$

### ABTS radical cation scavenging assay

The antioxidant scavenging activity against the 2,20-azino-bis-3-ethylbenzothiazoline-6-sulfonic acid radical cation (ABTS^•+^) was evaluated using a method previously described^94,96^ with some modifications. Briefly, 150 µL of an ethanolic dilution of the extracts (25, 125, 250, 500 µg mL^−1^) or 150 µL of the Trolox standard solution (5, 12.5, 25, 37.5 µmol L^−1^ in ethanol) were mixed in triplicate with 150 µL of an ABTS^•+^ working solution in ethanol (absorbance ~ 0.9, 734 nm). The mixture was incubated for 120 min and the absorbance was measured at 734 nm every 5 min (Multiskan GO 1.00.38, Thermo Scientific, Hudson, NH, USA). A control was prepared by replacing the ABTS^•+^ solution with ethanol. The antioxidant activity, expressed as a percentage of inhibition of the ABTS radical (IC40 substituted by IC50), was calculated using Eq. (2) (Abs_DPPH_^•^ substituted by Abs_ABTS_^•+^) and expressed in Trolox Equivalents (Eq. 3).

### Analysis of fatty acids by gas chromatography–mass spectrometry (GC–MS)

The fatty acid methyl esters (FAMEs) were prepared from total lipid extracts of *Chlorococcum amblystomatis* by transmethylation reaction using a methanolic solution of potassium hydroxide (2.0 M) according to the methodology previously described ^90^. A volume of 2.0 μL of a hexane solution containing FAMEs and 1.03 μg mL^−1^ of methyl nonadecanoate (Sigma, St. Louis, MO, USA) as internal standard was injected in a chromatography-mass spectrometry (GC–MS) (Agilent Technologies 8860 GC System, Santa Clara, CA, USA) equipped with a DB-FFAP column with the following specifications: 30 m long, 0.32 mm internal diameter, and 0.25 μm film thickness (J & W Scientific, Folsom, CA, USA). The GC equipment was connected to an Agilent 5977B Mass Selective Detector operating with electron impact ionization at 70 eV and a scanning range of *m/z* 50–550 (1 s cycle in a full scan mode). The following conditions were used: helium as carrier gas (constant flow 1.4 mL min^−1^), inlet temperature 220 °C, detector temperature 230 °C, injection volume 2 μL (splitless). The oven temperature was programmed as follows: 58 °C for 2 min, 25 °C min^−1^ to 160 °C, 2 °C min^−1^ to 210 °C, 30 °C min^−1^ to 225 °C (held for 20 min). The data acquisition software used was GCMS5977B/Enhanced MassHunter. Data were analysed using Agilent MassHunter Qualitative Analysis 10.0 software. The identification of FA was carried out by comparison of the MS spectrum with the NIST chemical database library and confirmed with the literature reports. Five independent replicates were injected.

#### Atherogenic, thrombogenic and hypocholesterolemic/hypercholesterolemic indices

The atherogenic, thrombogenic and hypocholesterolemic/hypercholesterolemic indices (h/H) were calculated as the content ratio of saturated fatty acids (SFA)/unsaturated FA, as monounsaturated fatty acids (MUFAs) and omega-3 and omega-6 PUFAs, using the following formula (Eqs. 4, 5 and 6), as proposed by Ulbricht and Southgate ^97^

4 $${\text{Atherogenic index (AI)}} = \frac{{{\text{[C12:0}} + {4*}\left( {\text{C14:0}} \right) + {\text{C16:0]}}}}{{{[}\sum {\text{MUFA}} + \sum \left( {{\text{n}} - {6}} \right){ + }\sum {\text{(n}} - {3)]}}}$$

5 $${\text{Thrombogenic index (TI)}} = \frac{{{\text{[C14:0}} + {\text{C16:0}} + {\text{C18:0]}}}}{{\left[ {{0}{\text{.5*}}\sum {\text{MUFA}} + {0}{\text{.5*}}\sum \left( {{\text{n}} - {6}} \right){ + 3*}\sum \left( {{\text{n}} - {3}} \right) + \left( {\frac{{\sum {\text{(n}} - {6)}}}{{\sum {\text{(n}} - {3)}}}} \right)} \right]}}$$

6 $${\text{hypocholesterolemic}}/{\text{Hypercholesterolemic}}\,\,{\text{ratio }}\left( {{\text{h}}/{\text{H}}} \right) = \frac{{{\text{[C18:1}}\omega {9} + {18:2}\omega {6} + {18:3}\omega {3} + {\text{C20:4}}\omega {6} + {\text{C20:5}}\omega {3]}}}{{{\text{[C14:0}} + {\text{C16:0]}}}}$$

### Polar lipidome analysis by hydrophilic interaction liquid chromatography coupled to high-resolution tandem mass spectrometry (HILIC–HR–MS/MS)

The polar lipidome was determined according to the methodology previously described^34,35^. The dried samples were dissolved in CH_2_Cl_2_ to a final concentration of 1 ug uL^-1^. From each sample, a volume of 10 µL (10 µg of lipid extract) was taken and transferred to an appropriate vial, followed by the addition of 86 µL of a solvent system composed of two mobile phases in a proportion of 90% v/v eluent B and 10% v/v eluent A and 4 µL of a mixture of internal standards (dMPC—0.02 μg, dMPE—0.02 μg, SM (17:0/d18:1)—0.02 μg, LPC—0.02 μg, dPPI—0.08 μg, dMPG—0.012 μg, dMPS—0.04 μg). The composition of eluent B was 60% v/v of acetronitrile, 40% v/v of methanol, and 5 mM of ammonium acetate and the composition of eluent A was 50% v/v of acetonitrile, 25% v/v of methanol, 25% v/v of water, and 5 mM ammonium acetate.

The lipids were separated by hydrophilic interaction liquid chromatography (HILIC) using a microbore Ascentis Si column (10 cm × 1.0 mm, 3 µm; Sigma-Aldrich) and a high performance-liquid chromatography (HPLC) system (Ultimate 3000 Dionex, Thermo Fisher Scientific, Bremen, Germany) with an autosampler coupled to the Q-Exactive hybrid quadrupole Orbitrap mass spectrometer (Thermo Fisher Scientific, Bremen, Germany). A 5 µL aliquot of each sample mixture was injected into the HPLC column, at a flow rate of 50 µL min^−1^ and a temperature of 35 °C. The following gradient was applied: 10% A (0–2 min), 10–90% A (2–15 min), 90% A (15–17 min). The mass spectrometer with Orbitrap technology was operated simultaneously in positive (electrospray voltage 3.0 kV) and negative (electrospray voltage − 2.7 kV) modes, with the following configuration: high resolution with 70,000, AGC target of 1 × 10^6^, capillary temperature 250 °C, and sheath gas flow of 15 U. The tandem mass spectrometry experiments were performed according to the following configuration: resolution of 17,500, AGC target of 1 × 10^5^, with one full scan mass spectrum and 10 data-dependent MS/MS scans. The cycles were repeated continuously throughout the experiments with the dynamic exclusion of 60 s and an intensity threshold of 1 × 10^4^. Normalized collision energy (CE) ranged between 25, 30, and 35 eV. Data acquisition was performed using the Xcalibur data system (V3.3, Thermo Fisher Scientific, USA). Five independent biological replicas were carried out.

### Data analysis

To identify the classes of the polar lipids in the lipid extracts acquired spectra were analysed using Xcalibur v3.3 (Thermo Fisher Scientific, USA). The identification was carried out according to a standard approach in our laboratory ^98^: this approach consists of the localization of the species according to the retention time of internal standards (information related to internal standards can be found in Supplementary Table S1), accurate mass measurements (5 ppm) and identification of recurring fragmentation patterns (Supplementary Figures S1–S13) and their comparison with those of internal standards and published information on fragmentation patterns^98–101^. After identification, the quantification of molecular species was carried out by integrating the chromatographic peaks using the MZmine v2.42 software. The software allows filtering and smoothing, peak detection, peak processing and assignment against an internal database ^102^. All peaks of raw intensity bellow 1 × 10^4^ were excluded.

Relative quantification was performed by exporting the values of the peak areas to a computer spreadsheet (Excel, Microsoft, Redmond, WA). To normalize the data, the peak areas of the extracted ion chromatograms (EIC) of each lipid molecular species were divided by the EIC peak areas of the selected internal standards. Relative abundances were calculated using dMPC, dMPE, SM(17:0/d18:1), LPC, dPPI, dMPG and dMPS as internal standards. To normalize DGTS and MGTS we used PE as internal standard, for SQDG we used PG as internal standard, and for MGDG, DGDG, DGMG and MGMG, we used Ceramide as internal standard. For clear visualization, normalized data was transformed in percentage. The normalized data was calculated as follows (Eq. 7):

7 $${\text{Relative quantification \% }} = \frac{{\frac{{\left( {\text{EIC lipid species}} \right)}}{{\left( {\text{EIC internal standard}} \right)}}{ }}}{{\sum {\text{Total Normalized EICs}}}} \times 100$$

The relative percentage of betaines, phospholipids and glycolipids was calculated as follows (Eq. 8):

8 $${\text{Relative quantification classes\% }} = \frac{{\sum {\text{Normalized EICs each class}}}}{{\sum {\text{Total Normalized EICs}}}} \times 100$$

The relative percentage of the distribution of omega-3 and omega-6 fatty acids across the different main classes of polar lipids (betaines, phospholipids and glycolipids) was calculated as follows (Eq. 9):

9 $${\text{Relative quantification of }}\omega - {3}/\omega - {\text{6 polar lipids }} = \,\,\, \sum {\text{Relative percentage of each species calculated in Equation }}7$$

Raw abundances, normalized data and relative quantification can be found on the additional spreadsheet (Supplementary file S1).

## Supplementary Information

Supplementary Information

## Data Availability

Raw datasets generated during this study are available from the corresponding authors upon reasonable request.
