# Supplementary material for: Polar lipidomic profile shows Chlorococcum amblystomatis as a promising source of value-added lipids
Source: Sci Rep. 2021 Feb 23;11:4355. doi: 10.1038/s41598-021-83455-y (PMC7902829; doi:10.1038/s41598-021-83455-y)
Supplement: Supplementary file 1 — Supplementary Information [file 41598_2021_83455_MOESM1_ESM.doc]

**Polar lipidomic profile shows Chlorococcum amblystomatis as a promising source of value-added lipids**

Tiago A. Conde^1^, Daniela Couto^1,2^,Tânia Melo^1,2^, Margarida Costa^3^, Joana Silva^3^, Maria R. Domingues^1,2^, Pedro Domingues^1^*

^1^Mass Spectrometry Centre, LAQV REQUIMTE, Department of Chemistry, University of Aveiro, Santiago University Campus, 3810-193 Aveiro, Portugal

^2^CESAM - Centre for Environmental and Marine Studies, Department of Chemistry, University of Aveiro, Santiago University Campus, 3810-193 Aveiro, Portugal

^3^Allmicroalgae Natural Products S.A., R&D Department, Rua 25 de Abril 19, 2445-287 Pataias, Portugal

*Corresponding authors: Pedro Domingues, p.domingues@ua.pt

**Supplementary table 1 –** Fragmentation pattern of internal standards (IS) used to identify the polar lipidome of *Chlorococcum amblystomatis* by HILIC-HR-ESI-MS and MS/MS. Assignment of polar lipids was confirmed by the presence of fragment ions and neutral losses of the polar heads, and identification of acyl side chains RCOO^-^ fragment ions (in negative ion mode) and through neutral losses. All ions are assigned with their, observed mass, calculated mass, mass error, polar head and fatty acyl chains *m/z*.

| **Lipid species (C:N)** | **Calculated *m/z*** | **Observed *m/z*** | **Error (ppm)** | **Fatty acyl chains (C:N)** | **Polar head *m/z*** | **Fatty acyl chains *m/z*** |
| --- | --- | --- | --- | --- | --- | --- |
| **Internal standards as [M-H]-** | | | | | | |
| PE(28:0) | 634.4448 | 634.4455 | **1.1** | 14:0/14:0 | 140.0106 | 227.2016 |
| PG(28:0) | 665,4394 | 665,4396 | **0.3** | 14:0/14:0 | 171.0052 | 227.2015 |
| PI(32:0) | 809.5180 | 809.5161 | **-2.3** | 16:0/16:0 | 241.0121 | 255.2331 |
| **Internal standards as [M+CH3COO]-** | | | | | | |
| LPC(19:0) | 596.3927 | 596.3945 | **3.0** | 19:0 | 168.0423 | 227.2015 |
| PC(28:0) | 736.5129 | 736.5137 | **1.1** | 14:0/14:0 | 168.0423 | 227.2015 |
| **Internal standards as [M+H]+** | | | | | | |
| LPC(19:0) | 538.3873 | 538.3874 | **0.12** | 19:00 | 184.0731 | - |
| PC(28:0) | 678.5074 | 678.5074 | **0.0** | 14:0/14:0 | 184.0732 | -228.204 |
| PE(28:0) | 636.4604 | 636.4604 | **0.0** | 14:0/14:0 | -141.0127 | - |
| Cer(d35:1) | 552.5356 | 552.5352 | **-0.7** | 14:0/14:0 | 264.2683 | - |

- the fragment/neutral loss was not observed.

**Supplementary table 2 –** Polar lipid species identified in the total lipid extract of Chlorococcum amblystomatis by HILIC-HR-ESI-MS and MS/MS. Assignment of polar lipids was confirmed by the presence of fragment ions and neutral losses of the polar heads. and identification of acyl side chains RCOO^-^ fragment ions (in negative ion mode) and through neutral losses. All ions are assigned with their. observed mass. calculated mass. mass error. polar head and fatty acyl chains *m/z*.

| **Lipid species (C:N)** | **Calculated *m/z*** | **Observed *m/z*** | **Error (ppm)** | **Fatty acyl chains (C:N)** | **Polar head *m/z*** | **Fatty acyl chains *m/z*** |  | | | | |
| --- | --- | --- | --- | --- | --- | --- | --- | --- | --- | --- | --- |
| **LPC identified as [M+H]^+^** | | | | | | |  | |  | | |
| LPC14:0) | 468.309 | 468.3086 | **-0.9** | ****** | **-** | - |  | | | | |
| LPC(16:0) | 496.3403 | 496.3404 | **0.2** | ** | - | - |  | | | | |
| LPC(16:1) | 494.3247 | 494.3244 | **-0.5** | 16:01 | 184.0732 | 253.2174 |  | | | | |
| LPC(16:2) | 492.309 | 492.3083 | **-1.5** | ****** | **-** | - |  | | | | |
| LPC(18:1) | 522.356 | 522.3561 | **0.3** | 18:01 | 184.0731 | 281.2487 |  | | | | |
| LPC(18:2) | 520.3403 | 520.3402 | **-0.2** | 18:02 | 184.0734 | 279.2335 |  | | | | |
| LPC(18:3) | 518.3247 | 518.3233 | **-2.6** | * | 184.0733 | - |  | | | | |
| LPC(18:4) | 516.309 | 516.3067 | **-4.5** | ***** | 184.0602 | - |  | | | | |
| LPC(20:3) | 546.356 | 546.3560 | **0.1** | ****** | **-** | - |  | | | | |
| LPC(20:4) | 544.3403 | 544.3398 | **-0.9** | ** | - | - |  | | | | |
| LPC(20:5) | 542.3247 | 542.3241 | **-1.0** | * | 184.0729 | - |  | | | | |
| **PC identified as [M+H]^+^** | | | | | | |  | | | |  |
| PC(28:1) | 676.4917 | 676.4905 | **-1.8** | * | 184.0731 | - |  | | | | |
| PC(30:0) | 706.5387 | 706.5371 | **-2.2** | ** | - | - |  | | | | |
| PC(30:1) | 704.523 | 704.5250 | **2.8** | 14:0-16:1 | 184.0732 | 227.2015-253.2174 |  | | | | |
| PC(30:3) | 700.4917 | 700.4895 | **-3.2** | * | 184.0728 | - |  | | | | |
| PC(32:1) | 732.5543 | 732.5539 | **-0.6** | 16:0-16:1 | - | 255.2312-253.2159 |  | | | | |
| PC(32:2) | 730.5387 | 730.5384 | **-0.4** | 16:1-16:1 | 184.0731 | 253.2173 |  | | | | |
| PC(32:3) | 728.523 | 728.5230 | **0.0** | 16:1-16:2 | 184.0732 | 253.2176-251.2021 |  | | | | |
| PC(32:4) | 726.5074 | 726.5058 | **-2.2** | * | 184.0732 | - |  | | | | |
| PC(32:5) | 724.4917 | 724.4908 | **-1.3** | ** | - | - |  | | | | |
| PC(34:1) | 760.5856 | 760.5842 | **-1.9** | 16:0-18:1 | 184.0735 | 255.2332-281.2489 |  | | | | |
|  |  |  |  | 16:1-18:0 |  | 253.2170-283.2522 |  | | | | |
| PC(34:2) | 758.57 | 758.5696 | **-0.5** | 16:1-18:1 | 184.0732 | 253.2174-281.2487 |  | | | | |
|  |  |  |  | 16:0-18:2 |  | 255.2331-279.2332 |  | | | | |
| PC(34:3) | 756.5543 | 756.5542 | **-0.2** | 16:1-18:2 | 184.0733 | 253.2173-279.2331 |  | | | | |
| PC(34:4) | 754.5387 | 754.5371 | **-2.1** | 16:1-18:3 | 184.0732 | 253.2173-277.2175 |  | | | | |
|  |  |  |  | 16:2-18:2 |  | 251.2012-279.2328 |  | | | | |
| PC(34:5) | 752.523 | 752.5210 | **-2.7** | * | 184.0732 | - |  | | | | |
| PC(34:6) | 750.5074 | 750.5064 | **-1.3** | * | 184.0736 | - |  | | | | |
| PC(34:7) | 748.4917 | 748.4899 | **-2.4** | ****** | **-** | - |  | | | | |
| PC(36:2) | 786.6013 | 786.6004 | **-1.1** | ** | - | - |  | | | | |
| PC(36:3) | 784.5856 | 784.5850 | **-0.8** | 18:1-18:2 | 184.0732 | 281.2488-279.2330 |  | | | | |
| PC(36:4) | 782.57 | 782.5682 | **-2.3** | 18:2-18:2 | 184.0733 | 279.2332 |  | | | | |
|  |  |  |  | 16:0-20:4 |  | 255.2336-303.2339 |  | | | | |
| PC(36:5) | 780.5543 | 780.5528 | **-2.0** | 18:2-18:3 | 184.0733 | 279.2334-277.2174 |  | | | | |
|  |  |  |  | 16:0-20:5 |  | 255.2333-301.2172 |  | | | | |
|  |  |  |  | 16:1-20:4 |  | 253.2172-303.2336 |  | | | | |
| PC(36:6) | 778.5387 | 778.5379 | **-1.0** | 16:1-20:5 | 184.0734 | 253.2179-301.2174 |  | | | | |
| PC(36:7) | 776.523 | 776.5212 | **-2.4** | * | 184.0731 | - |  | | | | |
| PC(38:5) | 808.5856 | 808.5843 | **-1.6** | 18:1-20:4 | 184.0735 | 281.2485-303.2327 |  | | | | |
| PC(38:6) | 806.57 | 806.5690 | **-1.2** | 18:2-20:4 | 184.0732 | 279.2331-303.2339 |  | | | | |
|  |  |  |  | 18:1-20:5 |  | 281.2488-301.2184 |  | | | | |
| PC(38:7) | 804.5543 | 804.5525 | **-2.3** | 18:2-20:5 | 184.0731 | 279.2327-301.2180 |  | | | | |
| PC(38:8) | 802.5387 | 802.5351 | **-4.5** | ****** | **-** | - |  | | | | |
| PC(38:9) | 800.523 | 800.5204 | **-3.3** | ***** | 184.0732 | - |  | | | | |
| PC(40:10) | 826.5387 | 826.5369 | **-2.2** | * | 184.0732 | - |  | | | | |
| PC(40:5) | 836.6169 | 836.6180 | **1.3** | ***** | 184.0732 | - |  | | | | |
| PC(40:7) | 832.5856 | 832.5831 | **-3.0** | ****** | **-** | - |  | | | | |
| PC(40:8) | 830.57 | 830.5674 | **-3.1** | * | 184.0732 | - |  | | | | |
| PC(40:9) | 828.5543 | 828.5520 | **-2.8** | * | 184.0731 | - |  | | | | |
| **LPE identified as [M+H]^+^ - polar head as neutral loss** | | | | | | | |  | |  | |
| LPE(14:0) | 426.2621 | 426.2615 | **-1.3** | ** | - | - |  | | | | |
| LPE(16:0) | 454.2934 | 454.2928 | **-1.2** | 16:00 | -141.0203 | 255.2333 |  | | | | |
| LPE(16:1) | 452.2777 | 452.2774 | **-0.7** | * | -141.0214 | - |  | | | | |
| LPE(16:4) | 446.2308 | 446.2303 | **-1.0** | ** | - | - |  | | | | |
| LPE(18:1) | 480.309 | 480.3092 | **0.4** | 18:01 | -141.0191 | 281.2491 |  | | | | |
| LPE(18:2) | 478.2934 | 478.2926 | **-1.6** | ** | - | - |  | | | | |
| LPE(18:3) | 476.2777 | 476.2774 | **-0.7** | * | -141.0204 | - |  | | | | |
| LPE(18:4) | 474.2621 | 474.2618 | **-0.6** | 18:04 | -141.0201 | 275.2033 |  | | | | |
| LPE(20:5) | 500.2777 | 500.2781 | **0.8** | ** | - | - |  | | | | |
| **PE identified as [M+H]^+^ - polar head as neutral loss** | | | | | | | |  | |  | |
| PE(30:1) | 662.4761 | 662.4754 | **-1.0** | 14:0-16:1 | -141.0195 | 227.201-253.217 | 141.019097 | | | | |
| PE(30:3) | 658.4448 | 658.4422 | **-3.9** | * | -141.0205 | - |  | | | | |
| PE(32:1) | 690.5074 | 690.5058 | **-2.3** | * | -141.0193 | - |  | | | | |
| PE(32:2) | 688.4917 | 688.4925 | **1.1** | 16:1-16:1 | -141.0223 | 253.2172 |  | | | | |
| PE(32:4) | 684.4604 | 684.4593 | **-1.7** | 16:0-16:4 | -141.0189 | 255.2335-247.1697 |  | | | | |
| PE(34:1) | 718.5387 | 718.5353 | **-4.7** | 16:0-18:1 | -141.0159 | 255.2327-281.25 |  | | | | |
| PE(34:2) | 716.523 | 716.5219 | **-1.6** | 16:1-18:1 | -141.0199 | 253.2173-281.249 |  | | | | |
|  |  |  |  | 16:0-18:2 |  | 255.2338-279.2331 |  | | | | |
| PE(34:3) | 714.5074 | 714.5044 | **-4.2** | * | -141.0181 | - |  | | | | |
| PE(34:4) | 712.4917 | 712.4901 | **-2.3** | 16:0-18:4 | -141.0181 | 255.233-275.2027 |  | | | | |
| PE(34:5) | 710.4761 | 710.4754 | **-1.0** | 16:1-18:4 | -141.0196 | 253.2181-275.2028 |  | | | | |
| PE(36:2) | 744.5543 | 744.5535 | **-1.1** | 18:1-18:1 | -141.0201 | 281.2497 |  | | | | |
| PE(36:5) | 738.5074 | 738.5075 | **0.2** | 18:1-18:4 | -141.021 | 281.2486-275.2019 |  | | | | |
| PE(36:6) | 736.4917 | 736.4917 | **0.0** | * | -141.0222 | - |  | | | | |
| **PG identified as [M−H]^−^** | | | | | | | |  | |  | |
| PG(30:0) | 693.4707 | 693.4700 | **-1.0** | 14:0-16:0 | - | 227.2016-255.2342 | 804184.4242 | | | | |
| PG(30:1) | 691.455 | 691.4545 | **-0.7** | ** | - | - | 3833963.381 | | | | |
| PG(32:0) | 721.502 | 721.5026 | **0.8** | ** | - | - | 47670054.51 | | | | |
| PG(32:1) | 719.4863 | 719.4866 | **0.4** | 16:1-16:0 | 171.0053 | 255.2332-253.2175 | 63258812.52 | | | | |
|  |  |  |  | 14:0-18:1 |  | 227.2014-281.2489 | 6665800.124 | | | | |
| PG(32:2) | 717.4707 | 717.4703 | **-0.6** | 16:1-16:1 | 171.0056 | 253.2173 | 127190552.2 | | | | |
| PG(34:1) | 747.5176 | 747.5181 | **0.7** | 16:0-18:1 | 171.0055 | 255.2331-281.2489 | 145485481.7 | | | | |
| PG(34:2) | 745.502 | 745.5011 | **-1.2** | ** | - | - | 498042247.2 | | | | |
| PG(34:3) | 743.4867 | 743.4868 | **0.1** | 16:0-18:3 | 171.0058 and 227.0326 | 255.233-277.2174 | 168253515.4 | | | | |
| PG(34:4) | 741.4707 | 741.4711 | **0.5** | 16:0-18:4 | 171.0055 and 227.0323 | 255.2327-275.2026 | 2985003.326 | | | | |
|  |  |  |  | 16:1-18:3 |  | 253.2173-277.2175 | 10560899.36 | | | | |
| PG(34:5) | 739.455 | 739.4534 | **-2.2** | 14:0-20:5 | - | 227.2014-301.2179 | 356914246.6 | | | | |
|  |  |  |  | 16:2-18:3 |  | 251.2023-277.2174 | 192363075 | | | | |
| PG(36:2) | 773.5333 | 773.5337 | **0.5** | 18:1-18:1 | 171.0053 and 227.0329 | 281.2488 | 110603985.9 | | | | |
| PG(36:5) | 767.4864 | 767.4863 | **-0.1** | 16:0-20:5 | 171.006 | 255.2329-301.2171 | 4026676.772 | | | | |
| PG(36:6) | 765.4707 | 765.4715 | **1.0** | 16:1-20:5 | 171.0056 abd 227.0326 | 253.2174-301.2177 | 123709544.5 | | | | |
| PG(38:5) | 795.5176 | 795.5197 | **2.6** | ** | - | - | 278424310.1 | | | | |
| PG(32:2-OH) | 733.4656 | 733.4653 | **-0.4** | (16:0-OH)-16:2 | 171.0058 and 227.0328 | 271.2281-251.202 | 42248993.59 | | | | |
| PG(34:1-OH) | 763.5125 | 763.5115 | **-1.3** | (18:0-OH)-16:1 | 171.0058 | 299.26-253.2175 | 14398067.93 | | | | |
| PG(34:2-OH) | 761.4969 | 761.4971 | **0.2** | (18:0-OH)-16:2 | 171.0057 and 227.0325 | 299.2595-251.2016 | 4515826.281 | | | | |
| PG(34:3-OH) | 759.4812 | 759.4808 | **-0.5** | (18:3-OH)-16:0 | 171.0058 and 227.0327 | 293.2127-255.2331 | 71114438.68 | | | | |
|  |  |  |  | (18:2-OH)-16:1 |  | 295.2269-253.217 | 18194136.21 | | | | |
|  |  |  |  | (18:1-OH)-16:2 |  | 297.2437-251.2017 |  | | | | |
|  |  |  |  | (18:0-OH)-16:3 |  | 299.2595- - |  | | | | |
| PG(34:4-OH) | 757.4656 | 757.4653 | **-0.4** | (18:4-OH)-16:0 | 171.0056 and 227.0329 | 291.1951-255.2334 |  | | | | |
|  |  |  |  | (18:3-OH)-16:1 |  | 293.2121-253.2172 |  | | | | |
|  |  |  |  | (18:2-OH)-16:2 |  | 295.2276-251.2017 |  | | | | |
| PG(34:5-OH) | 755.4499 | 755.4480 | **-2.5** | (18:3-OH)-16:2 | 227.0318 | 293.2131-251.2021 |  | | | | |
|  |  |  |  | (18:4-OH)-16:1 |  | 291.1961- - |  | | | | |
| PG(36:5-OH) | 783.4812 | 783.4828 | **2.0** | (16:0-OH)-20:5 | 171.0055 and 227.0327 | 271.228-301.2174 |  | | | | |
|  |  |  |  | (20:5-OH)-16:0 |  | 317.2121-255.233 |  | | | | |
| PG(36:6-OH) | 781.4656 | 781.4665 | **1.1** | (20:5-OH)-16:1 | 171.0057 and 227.0325 | 317.2122-253.2177 |  | | | | |
| **PI identified as [M−H]^−^** | | | | | | | |  | |  | |
| PI(32:1) | 807.5024 | 807.5018 | **-0.7** | 16:0-16:1 | 241.012 | 255.2331-253.2176 |  | | | | |
| PI(34:1) | 835.5318 | 835.5302 | **-1.9** | 16:0-18:1 | 241.017 | 255.233-281.2487 |  | | | | |
| **PI-Cer identified as [M−H] −** | | | | | | | |  | |  | |
| PI-Cer(d18:1/14:0) | 750.4921 | 750.4921 | **0.0** | * | 241.0118 and 259.0229 | - |  | | | | |
| **MGTS identified as [M+H]^+^** | | | | | | | |  | | | |
| MGTS(14:0) | 446.3482 | 446.3479 | **-0.6** | 14:00 | 236.1494 | 236.1494 |  | | | | |
| MGTS(14:1) | 444.3325 | 444.3323 | **-0.5** | 14:01 | 236.1483 | 236.1483 |  | | | | |
| MGTS(16:0) | 474.3795 | 474.3793 | **-0.4** | 16:00 | 236.1489 | 236.1489 |  | | | | |
| MGTS(16:1) | 472.3638 | 472.3635 | **-0.7** | 16:01 | 236.1489 | 236.1489 |  | | | | |
| MGTS(16:2) | 470.3482 | 470.3485 | **0.7** | 16:02 | 236.1492 | 236.1492 |  | | | | |
| MGTS(16:3) | 468.3325 | 468.3335 | **2.1** | ** | - | - |  | | | | |
| MGTS(16:4) | 466.3169 | 466.3167 | **-0.4** | 16:04 | 236.1490 | 236.1490 |  | | | | |
| MGTS(18:0) | 502.4108 | 502.4115 | **1.5** | 18:00 | 236.1483 | 236.1483 |  | | | | |
| MGTS(18:1) | 500.3951 | 500.3948 | **-0.6** | 18:01 | 236.1492 | 236.1492 |  | | | | |
| MGTS(18:2) | 498.3795 | 498.3789 | **-1.1** | ** | - | - |  | | | | |
| MGTS(18:3) | 496.3638 | 496.3636 | **-0.4** | ** | - | - |  | | | | |
| MGTS(18:4) | 494.3482 | 494.3478 | **-0.7** | 18:04 | 236.1491 | 236.1491 |  | | | | |
| MGTS(18:5) | 492.3325 | 492.3318 | **-1.5** | ** | ** | - |  | | | | |
| MGTS(20:0) | 530.4421 | 530.4402 | **-3.5** | 20:00 | 236.1494 | 236.1494 |  | | | | |
| MGTS(20:4) | 522.3795 | 522.3798 | **0.6** | ** | - | - |  | | | | |
| MGTS(20:5) | 520.3638 | 520.3637 | **-0.2** | 20:05 | 236.1494 | 236.1494 |  | | | | |
| **DGTS identified as [M+H]^+^** | | | | | | | |  | | | |
| DGTS(28:1) | 654.5309 | 654.5325 | **2.4** | ****** | - | - |  | | | | |
| DGTS(30:0) | 684.5778 | 684.5759 | **-2.8** | 16:0-14:0 | 236.1486 | *446.3463-474.3781* |  | | | | |
| DGTS(30:1) | 682.5622 | 682.5619 | **-0.4** | 16:1-16:0 | 236.1487 | 428.3366-- |  | | | | |
| DGTS(30:2) | 680.5465 | 680.5444 | **-3.1** | 16:2-14:0 | 236.1497 | *446.3469*-- |  | | | | |
| DGTS(30:3) | 678.5309 | 678.5306 | **-0.4** | ** | - | - |  | | | | |
| DGTS(30:4) | 676.5152 | 676.5151 | **-0.1** | 16:4-16:0 | 236.1491 | 428.3381-- |  | | | | |
| DGTS(30:5) | 674.4996 | 674.5002 | **0.9** | 16:4-14:1 | 236.1492 | 426.3220-- |  | | | | |
| DGTS(32:0) | 712.6091 | 712.6067 | **-3.4** | 16:0-16:0 | 236.1488 | *474.3786--* |  | | | | |
| DGTS(32:1) | 710.5935 | 710.5933 | **-0.3** | 16:1-16:0 | 236.149 | 456.3677-454.3518 |  | | | | |
| DGTS(32:2) | 708.5778 | 708.5772 | **-0.8** | 16:0-16:2 | 236.1489 | 452.3387-456.3659 |  | | | | |
|  |  |  |  | 16:1-16:1 |  | 454.3512-454.3512 |  | | | | |
| DGTS(32:3) | 706.5622 | 706.5610 | **-1.7** | 16:3-16:0 | 236.1489 | 456.368-450.3206 |  | | | | |
| DGTS(32:4) | 704.5465 | 704.5464 | **-0.1** | 14:0-18:4 | 236.1489 | 476.3381-428.3385 |  | | | | |
|  |  |  |  | 16:4-16:0 |  | 456.3675-448.305 |  | | | | |
|  |  |  |  | 16:3-16:1 |  | *472.3615*-*468.3299* |  | | | | |
| DGTS(32:5) | 702.5309 | 702.5306 | **-0.4** | ** | - | - |  | | | | |
| DGTS(32:6) | 700.5152 | 700.5152 | **0.0** | 16:4-16:2 | 236.1489 | *470.3483-466.3201* |  | | | | |
|  |  |  |  | 16:3-16:3 |  | *468.3318-468.3318* |  | | | | |
| DGTS(32:7) | 698.4996 | 698.4993 | **-0.4** | 16:4-16:3 | 236.1488 | 450.3196-448.3041 |  | | | | |
| DGTS(32:8) | 696.4839 | 696.4844 | **0.7** | 16:4-16:4 | 236.1492 | 448.3054-448.3054 |  | | | | |
| DGTS(34:1) | 738.6248 | 738.6251 | **0.4** | 16:0-18:1 | 236.1488 | *500.3941*-456.3666 |  | | | | |
| DGTS(34:2) | 736.6091 | 736.6056 | **-4.8** | 16:1-18:1 | 236.1489 | *500.3947-472.3627* |  | | | | |
|  |  |  |  | 18:2-16:0 |  | *474.3785--* |  | | | | |
|  |  |  |  | 20:2-14:0 |  | 428.3361-- |  | | | | |
| DGTS(34:3) | 734.5935 | 734.5929 | **-0.8** | 16:2-18:1 | 236.1488 | 482.383-- |  | | | | |
|  |  |  |  | 16:1-18:2 |  | 480.3665-454.3531 |  | | | | |
|  |  |  |  | 20:3-14:0 |  | 428.336-- |  | | | | |
| DGTS(34:4) | 732.5778 | 732.5776 | **-0.3** | 18:4-16:0 | 236.149 | 456.3677-476.3368 |  | | | | |
|  |  |  |  | 18:3-16:1 |  | 454.353-478.3526 |  | | | | |
| DGTS(34:5) | 730.5622 | 730.5616 | **-0.8** | 18:4-16:1 | 236.1489 | 454.3516-476.3354 |  | | | | |
|  |  |  |  | 18:1-16:4 |  | 448.3058-482.3839 |  | | | | |
|  |  |  |  | 18:2-16:3 |  | 468.3289- |  | | | | |
| DGTS(34:5-OH) | 746.5571 | 746.5575 | **0.5** | (16:4-OH)-18:1 | 236.1487 | *500.3944-464.3001* |  | | | | |
|  |  |  |  | (16:1-OH)-18:4 |  | *494.3464--* |  | | | | |
|  |  |  |  | (16:0-OH)-18:5 |  | *492.3341--* |  | | | | |
| DGTS(34:6) | 728.5465 | 728.5458 | **-1.0** | ** | - | - |  | | | | |
| DGTS(34:7) | 726.5309 | 726.5306 | **-0.4** | 16:4-18:3 | 236.1488 | 478.3514-448.3044 |  | | | | |
|  |  |  |  | 16:3-18:4 |  | *494.3474-468.3306* |  | | | | |
| DGTS(34:7-OH) | 742.5258 | 742.5258 | **0.0** | (16:4-OH)-18:3 | 236.1492 | 478.3511-464.3009 |  | | | | |
|  |  |  |  | (16:3-OH)-18:4 |  | 476.3352-466.3148 |  | | | | |
| DGTS(34:8) | 724.5152 | 724.5149 | **-0.5** | 16:4-18:4 | 236.1489 | 476.3361-448.3054 |  | | | | |
| DGTS(36:10) | 748.5152 | 748.5140 | **-1.6** | 18:5-18:5 | 236.1489 | *492.3319-492.3319* |  | | | | |
| DGTS(36:4) | 760.6091 | 760.6098 | **0.9** | 16:0-20:4 | 236.1489 | 504.3693-*474.3784* |  | | | | |
|  |  |  |  | 18:3-18:1 |  | 482.3829-478.3513 |  | | | | |
|  |  |  |  | 18:2-18:2 |  | 480.3666-480.3666 |  | | | | |
|  |  |  |  | 20:3-16:1 |  | 454.3524-*524.3929* |  | | | | |
|  |  |  |  | 20:2-16:2 |  | 452.3382-- |  | | | | |
|  |  |  |  | 20:1-16:3 |  | 450.3216-- |  | | | | |
|  |  |  |  | 20:0-16:4 |  | 448.3063-*530.444* |  | | | | |
| DGTS(36:5) | 758.5935 | 758.5933 | **-0.3** | 18:4-18:1 | 236.1489 | 482.3837-*494.3464* |  | | | | |
|  |  |  |  | 20:5-16:0 |  | *474.3773*-502.3521 |  | | | | |
|  |  |  |  | 20:4-16:1 |  | *472.3625*-*522.377* |  | | | | |
|  |  |  |  | 20:3-16:2 |  | 452.3378-- |  | | | | |
|  |  |  |  | 20:2-16:3 |  | *468.3301*-- |  | | | | |
|  |  |  |  | 20:1-16:4 |  | 448.3046-- |  | | | | |
|  |  |  |  | 16:1-20:4 |  | *522.377*-454.3525 |  | | | | |
| DGTS(36:6) | 756.5778 | 756.5775 | **-0.4** | 16:1-20:5 | 236.1489 | 502.3538-454.3522 |  | | | | |
|  |  |  |  | 20:3-16:3 |  | *468.3307*-- |  | | | | |
|  |  |  |  | 18:2-18:4 |  | *494.3482*-480.3671 |  | | | | |
|  |  |  |  | 18:3-18:3 |  | 478.3518-478.3518 |  | | | | |
| DGTS(36:7) | 754.5622 | 754.5621 | **-0.1** | 18:4-18:3 | 236.1489 | *496.3624-494.3466* |  | | | | |
| DGTS(36:8) | 752.5465 | 752.5462 | **-0.4** | 18:4-18:4 | 236.149 | *494.3474-494.3474* |  | | | | |
| DGTS(36:9) | 750.5309 | 750.5281 | **-3.7** | 18:5-18.4 | 236.1487 | *494.3465-492.3323* |  | | | | |
| DGTS(38:10) | 776.5465 | 776.5464 | **-0.2** | 20:5-18:5 | 236.149 | *492.3327--* |  | | | | |
| DGTS(38:7) | 782.5935 | 782.5929 | **-0.8** | 20:5-18:2 | 236.1489 | 480.3680-502.3507 |  | | | | |
| DGTS(38:8) | 780.5778 | 780.5743 | **-4.5** | 20:5-18:3 | 236.1487 | 478.3515-502.3511 |  | | | | |
| DGTS(40:10) | 804.5778 | 804.5772 | **-0.7** | 20:5-20:5 | 236.1489 | 502.3518-502.3518 |  | | | | |
| DGTS(40:9) | 806.5935 | 806.5905 | **-3.7** | 20:4-20:5 | 236.1489 | 502.3517-- |  | | | | |
| **MGMG identified as [M+NH_4_]^+^** | | | | | | | |  | |  | |
| MGMG(14:0) | 482.3329 | 482.3324 | **-1.1** | 14:00 | 197.0906 | 285.2418 |  | | | | |
| MGMG(16:0) | 510.3642 | 510.3639 | **-0.6** | 16:00 | 197.0906 | 313.2733 |  | | | | |
| MGMG(16:1) | 508.3486 | 508.3482 | **-0.7** | 16:01 | 197.0904 | 311.2578 |  | | | | |
| MGMG(16:3) | 504.3173 | 504.3174 | **0.3** | ** | - | - |  | | | | |
| MGMG(16:4) | 502.3016 | 502.3013 | **-0.6** | 16:04 | 197.0909 | 305.2104 |  | | | | |
| MGMG(16:4-OH) | 518.2965 | 518.2963 | **-0.4** | 16:4-OH | 197.0898 | 321.2065 |  | | | | |
| MGMG(18:1) | 536.3799 | 536.3793 | **-1.0** | 18:01 | 197.0902 | 339.2891 |  | | | | |
| MGMG(18:3) | 532.3486 | 532.3485 | **-0.1** | 18:03 | 197.0905 | 335.258 |  | | | | |
| MGMG(18:4) | 530.3329 | 530.3330 | **0.2** | * | 197.0869 | - |  | | | | |
| MGMG(20:4) | 558.3642 | 558.3636 | **-1.1** | ** | - | - |  | | | | |
| MGMG(20:5) | 556.3486 | 556.3482 | **-0.6** | ** | - | - |  | | | | |
| **MGDG identified as [M+NH_4_]^+^** | | | | | | | |  | |  | |
| MGDG(30:0) | 720.5625 | 720.5593 | **-4.4** | ** | - | - |  | | | | |
| MGDG(30:1) | 718.5464 | 718.5466 | **0.3** | 16:1-14:0 | 197.0898 | 311.2576-285.2420 |  | | | | |
| MGDG(32:0) | 748.5939 | 748.5910 | **-3.8** | 16:0-16:0 | 197.0948 |  |  | | | | |
| MGDG(32:1) | 746.5777 | 746.5780 | **0.4** | 16:1-16:0 | 197.0919 | 311.2571-313.2732 |  | | | | |
| MGDG(32:2) | 744.5626 | 744.5624 | **-0.3** | 16:1-16:1 | 197.0910 | 311.2576-311.2576 |  | | | | |
|  |  |  |  | 14:0-18:2 |  | 285.2419-337.2725 |  | | | | |
|  |  |  |  | 16:0-16:2 |  | 313.2738-- |  | | | | |
| MGDG(32:3) | 742.5464 | 742.5467 | **0.4** | ** | - | - |  | | | | |
| MGDG(32:4) | 740.5307 | 740.5305 | **-0.3** | ** | - | - |  | | | | |
| MGDG(32:5) | 738.5156 | 738.5158 | **0.2** | ** | - | - |  | | | | |
| MGDG(32:6) | 736.4994 | 736.4999 | **0.7** | ** | - | - |  | | | | |
| MGDG(32:7) | 734.4843 | 734.4835 | **-1.1** | ** | - | - |  | | | | |
| MGDG(32:8) | 732.4687 | 732.4686 | **-0.1** | 16:4-16:4 | - | 305.2104-305.2104 |  | | | | |
| MGDG(34:1) | 774.6090 | 774.6073 | **-2.2** | ** | - | -- |  | | | | |
| MGDG(34:2) | 772.5933 | 772.5926 | **-0.9** | 18:2-16:0 | 197.0910 | 337.2722-313.2730 |  | | | | |
| MGDG(34:3) | 770.5782 | 770.5782 | **0.0** | 18:3-16:0 | 197.0930 | 335.2575-313.2733 |  | | | | |
|  |  |  |  | 18:2-16:1 |  | 337.2728-311.2576 |  | | | | |
| MGDG(34:4) | 768.5626 | 768.5645 | **2.5** | 16:2-18:2 | 197.0979 | 309.2404-- |  | | | | |
|  |  |  |  | 16:1-18:3 |  | 311.2575-335.2570 |  | | | | |
|  |  |  |  | 16:0-18:4 |  | 313.2732-333.2437 |  | | | | |
|  |  |  |  | 16:3-18:1 |  | --339.2891 |  | | | | |
|  |  |  |  | 16:4-18:0 |  | 305.2103-341.2986 |  | | | | |
| MGDG(34:5) | 766.5469 | 766.5474 | **0.6** | * | 197.0964 | - |  | | | | |
| MGDG(34:7) | 762.5156 | 762.5158 | **0.2** | 16:4-18:3 | 197.0924 | 305.2106-335.2575 |  | | | | |
|  |  |  |  | 18:4-16:3 |  | 333.2424-- |  | | | | |
| MGDG(34:8) | 760.5000 | 760.4999 | **-0.1** | ** | - | - |  | | | | |
| MGDG(36:3) | 798.6095 | 798.6055 | **-5.0** | ** | - | - |  | | | | |
| MGDG(36:4) | 796.5933 | 796.5892 | **-5.1** | ** | - | - |  | | | | |
| MGDG(36:5) | 794.5782 | 794.5778 | **-0.5** | 18:3-18:2 | 197.0897 | 335.2576-- |  | | | | |
|  |  |  |  | 18:1-18:4 |  | 339.2885-333.2422 |  | | | | |
|  |  |  |  | 20:5-16:0 |  | 359.2579-313.2731 |  | | | | |
|  |  |  |  | 16:4-20:2 |  | 305.2112-- |  | | | | |
|  |  |  |  | 16:1-20:4 |  | 311.2573-- |  | | | | |
| MGDG(36:6) | 792.5625 | 792.5623 | **-0.3** | 18:3-18:3 | 197.0841 | 335.2572-335.2572 |  | | | | |
|  |  |  |  | 16:1-20:5 |  | 311.2574-359.2574 |  | | | | |
|  |  |  |  | 16:4-20:2 |  | 305.2106-- |  | | | | |
| MGDG(36:7) | 790.5469 | 790.5470 | **0.1** | ** | - | - |  | | | | |
| MGDG(36:8) | 788.5313 | 788.5339 | **3.3** | ** | - | - |  | | | | |
| MGDG(38:6) | 820.5939 | 820.5931 | **-1.0** | 18:1-20:5 | - | 339.2894-- |  | | | | |
|  |  |  |  | 18:2-20:4 |  | 337.2734-- |  | | | | |
|  |  |  |  | 18:3-20:3 |  | 335.2573-- |  | | | | |
| MGDG(38:7) | 818.5782 | 818.5777 | **-0.6** | 20:5-18:2 | 197.0886 | 359.2585-337.2729 |  | | | | |
|  |  |  |  | 18:3-20:4 |  | 335.2579-- |  | | | | |
| MGDG(38:8) | 816.5626 | 816.5614 | **-1.4** | ** | - | - |  | | | | |
| MGDG(40:10) | 840.5626 | 840.5622 | **-0.4** | 20:5-20:5 | - | 359.2573-359.2573 |  | | | | |
| MGDG(40:8) | 844.5939 | 844.5916 | **-2.7** | 20:4-20:4 | - | 361.2754-361.2754 |  | | | | |
| MGDG(40:7) | 846.6095 | 846.6097 | **0.2** | ** | - | - |  | | | | |
| **DGMG identified as [M+NH_4_]^+^** | | | | | | | |  | |  | |
| DGMG(14:0) | 644.3857 | 644.3848 | **-1.4** | ** | - | - |  | | | | |
| DGMG(16:0) | 672.4170 | 672.4172 | **0.3** | 16:00 | 359.1441 | 313.2731 |  | | | | |
| DGMG(16:1) | 670.4014 | 670.4012 | **-0.3** | 16:01 | 359.1438 | 311.2574 |  | | | | |
| DGMG(16:2) | 668.3857 | 668.3854 | **-0.5** | ** | - | - |  | | | | |
| DGMG(16:3) | 666.3701 | 666.3699 | **-0.3** | ** | - | - |  | | | | |
| DGMG(16:4) | 664.3544 | 664.3541 | **-0.5** | ** | - | - |  | | | | |
| DGMG(18:1) | 698.4327 | 698.4327 | **0.0** | 18:01 | 359.144 | 339.2887 |  | | | | |
| DGMG(18:2) | 696.4170 | 696.4149 | **-3.0** | ** | - | - |  | | | | |
| DGMG(18:3) | 694.4014 | 694.4013 | **-0.1** | 18:03 | 359.144 | 335.2573 |  | | | | |
| DGMG(18:4) | 692.3857 | 692.3856 | **-0.2** | ** | - | - |  | | | | |
| DGMG(20:5) | 718.4014 | 718.4009 | **-0.7** | ** | - | - |  | | | | |
| **DGDG identified as [M+NH_4_]^+^** | | | | | | | |  | |  | |
| DGDG(30:0) | 882.6154 | 882.6111 | **-4.9** | ** | - | - |  | | | | |
| DGDG(30:1) | 880.5997 | 880.5990 | **-0.8** | 16:1-14:0 | 359.1428 | 311.2574-285.2420 |  | | | | |
| DGDG(32:1) | 908.6310 | 908.6306 | **-0.4** | 16:0-16:1 | 359.1438 | 313.2730-311.2574 |  | | | | |
| DGDG(32:2) | 906.6154 | 906.6151 | **-0.3** | 16:0-16:2 | 359.1438 | 313.2735-309.2388 |  | | | | |
|  |  |  |  | 16:1-16:1 |  | 311.2575-311.2575 |  | | | | |
| DGDG(32:3) | 904.5997 | 904.6011 | **1.5** | 18:3-14:0 | 359.1452 | 335.2573-285.2418 |  | | | | |
|  |  |  |  | 16:0-16:3 |  | 313.2729-307.2259 |  | | | | |
|  |  |  |  | 16:1-16:2 |  | 311.2573-309.2416 |  | | | | |
| DGDG(32:4) | 902.5841 | 902.5846 | **0.6** | 18:3-14:1 | 359.1442 | 335.257-283.2263 |  | | | | |
|  |  |  |  | 16:0-16:4 |  | 313.2730-305.2108 |  | | | | |
|  |  |  |  | 14:0-18:4 |  | 285.2430-- |  | | | | |
| DGDG(32:5) | 900.5684 | 900.5678 | **-0.7** | 16:1-16:4 | - | 311.2577-305.21 |  | | | | |
| DGDG(32:6) | 898.5528 | 898.5512 | **-1.8** | ** | - | - |  | | | | |
| DGDG(34:1) | 936.6623 | 936.6615 | **-0.9** | 18:1-16:0 | 359.1434 | 339.2888-313.2730 |  | | | | |
| DGDG(34:2) | 934.6467 | 934.6443 | **-2.6** | 18:1-16:1 | 359.1421 | 339.2881-311.2574 |  | | | | |
|  |  |  |  | 18:2-16:0 |  | 337.2729-313.2731 |  | | | | |
| DGDG(34:3) | 932.6310 | 932.6309 | **-0.1** | 16:0-18:3 | 359.1435 | 313.2733-335.2577 |  | | | | |
|  |  |  |  | 18:1-16:2 |  | 339.2901-- |  | | | | |
| DGDG(34:4) | 930.6154 | 930.6143 | **-1.2** | 18:1-16:3 | 359.1427 | 339.2885-307.2260 |  | | | | |
|  |  |  |  | 18:2-16:2 |  | 337.2729--309.2417 |  | | | | |
|  |  |  |  | 18:3-16:1 |  | 335.2575-311.2574 |  | | | | |
|  |  |  |  | 18:4-16:0 |  | 333.2420-313.2729 |  | | | | |
|  |  |  |  | 14:0-20:4 |  | 285.2429-- |  | | | | |
| DGDG(34:5) | 928.5997 | 928.5991 | **-0.6** | 18:3-16:2 | 359.1443 | 335.2571-309.2415 |  | | | | |
|  |  |  |  | 18:2-16:3 |  | 337.2726-307.2266 |  | | | | |
|  |  |  |  | 18:1-16:4 |  | 339.2889-305.2108 |  | | | | |
|  |  |  |  | 16:1-18:4 |  | 311.2569-- |  | | | | |
|  |  |  |  | 14:0-20:5 |  | 285.2429-359.2569 |  | | | | |
| DGDG(34:6) | 926.5841 | 926.5820 | **-2.3** | ** | - | - |  | | | | |
| DGDG(34:7) | 924.5684 | 924.5684 | **0.0** | 18:3-16:4 | 359.1428 | 335.2574-305.2104 |  | | | | |
|  |  |  |  | 18:4-16:3 |  | 333.2414-307.2255 |  | | | | |
| DGDG(34:8) | 922.5528 | 922.5501 | **-2.9** | ** | - | - |  | | | | |
| DGDG(36:2) | 962.6780 | 962.6751 | **-3.0** | ** | - | - |  | | | | |
| DGDG(36:3) | 960.6623 | 960.6606 | **-1.8** | 18:3-18:0 | 359.1449 | 335.2575-- |  | | | | |
|  |  |  |  | 18:1-18:2 |  | 339.2897-- |  | | | | |
| DGDG(36:4) | 958.6467 | 958.6433 | **-3.5** | ** | - | - |  | | | | |
| DGDG(36:5) | 956.6310 | 956.6301 | **-0.9** | 20:5-16:0 | 359.1444 | 359.2571-313.2731 |  | | | | |
|  |  |  |  | 18:1-18:4 |  | 339.2888-333.2424 |  | | | | |
|  |  |  |  | 20:1-16:4 |  | 367.3203-305.2103 |  | | | | |
| DGDG(36:6) | 954.6154 | 954.6152 | **-0.2** | 18:3-18:3 | 359.1435 | 335.2575-335.2575 |  | | | | |
|  |  |  |  | 20:5-16:1 |  | 359.2577-311.2575 |  | | | | |
|  |  |  |  | 18:4-18:2 |  | 333.2417-337.2732 |  | | | | |
| DGDG(36:7) | 952.5997 | 952.5999 | **0.2** | 18:3-18:4 | - | 335.2568-- |  | | | | |
| DGDG(36:8) | 950.5841 | 950.5835 | **-0.6** | 18:4-18:4 | - | 333.2410-333.2410 |  | | | | |
| DGDG(38:6) | 982.6467 | 982.6421 | **-4.7** | ** | - | - |  | | | | |
| DGDG(38:7) | 980.6310 | 980.6312 | **0.2** | 18:2-20:5 | - | 337.2731-359.2565 |  | | | | |
| DGDG(40:10) | 1002.6154 | 1002.6148 | **-0.6** | 20:5-20:5 | - | 359.2577-359.2577 |  | | | | |
| DGDG(40:9) | 1004.6310 | 1004.6323 | **1.3** | ** | - | - |  | | | | |
| **SQDG identified as [M−H]^−^** | | | | | | | |  | |  | |
| SQDG(28:0) | 737.451 | 737.4492 | **-2.4** | ** | 225.0071 | - |  | | | | |
| SQDG(30:0) | 765.4823 | 765.4818 | **-0.6** | 16:0-14:0 | 225.0072 | 255.2332- - |  | | | | |
| SQDG(30:1) | 763.4666 | 763.4667 | **0.1** | 16:1-14:0 | 225.0072 | 253.2175- - |  | | | | |
| SQDG(32:0) | 793.5136 | 793.5135 | **-0.1** | ** | - | - |  | | | | |
| SQDG(32:1) | 791.4979 | 791.4982 | **0.3** | 16:0-16:1 | 225.0072 | 255.2348-253.2178 |  | | | | |
| SQDG(32:2) | 789.4823 | 789.4821 | **-0.2** | 16:1-16:1 | 225.007 | 253.2177 |  | | | | |
|  |  |  |  | 16:0-16:2 |  | 255.2342- - |  | | | | |
| SQDG(32:3) | 787.4666 | 787.4668 | **0.2** | 16:1-16:2 | 225.0072 | 253.2173- - |  | | | | |
|  |  |  |  | 16:0-16:3 |  | 255.2331- - |  | | | | |
|  |  |  |  | 18:1-14:1 |  | 281.249- - |  | | | | |
| SQDG(32:4) | 785.451 | 785.4517 | **0.9** | 16:0-16:4 | 225.0072 | 255.2324- - |  | | | | |
|  |  |  |  | 16:1-16:3 |  | 253.2175- - |  | | | | |
|  |  |  |  | 16:2-16:2 |  | 251.2017 |  | | | | |
| SQDG(34:0) | 821.5449 | 821.5446 | **-0.3** | 16:0-18:0 | 225.0072 | 255.2327- - |  | | | | |
| SQDG(34:1) | 819.5292 | 819.5296 | **0.5** | 16:0-18:1 | 225.0073 | 255.2332-281.249 |  | | | | |
| SQDG(34:3) | 815.4979 | 815.4982 | **0.3** | 16:0-18:3 | 225.0069 | 255..232- - |  | | | | |
|  |  |  |  | 18:1-16:2 |  | 281.25- - |  | | | | |
| SQDG(34:4) | 813.4823 | 813.4829 | **0.8** | 16:0-18:4 | 225.0075 | 255.2331- - |  | | | | |
|  |  |  |  | 16:1-18:3 |  | 253.2174-277.217 |  | | | | |
|  |  |  |  | 16:2-18:2 |  | 251.2014-279.233 |  | | | | |
|  |  |  |  | 18:1-16:3 |  | 281.2489- - |  | | | | |
|  |  |  |  | 20:4-14:0 |  | 303.2332- - |  | | | | |
| SQDG(36:0) | 849.5762 | 849.5761 | **-0.1** | 16:0-20:0 | 225.0073 | 255.2332- - |  | | | | |
| SQDG(36:3) | 843.5292 | 843.5287 | **-0.6** | ** | - | - |  | | | | |
| SQDG(36:4) | 841.5136 | 841.5135 | **-0.1** | 16:0-20:4 | 225.0069 | 255.2346- - |  | | | | |
| SQDG(36:5) | 839.4979 | 839.4987 | **0.9** | ** | - | - |  | | | | |
| SQDG(36:6) | 837.4823 | 837.4823 | **0.0** | 16:1-20:5 | 225.007 | 253.2175-301.2179 |  | | | | |
|  |  |  |  | 20:4-16:2 |  | 303.2311- - |  | | | | |
|  |  |  |  | 18:2-18:4 |  | 279.2317-275.2012 |  | | | | |
|  |  |  |  | 18:3-18:3 |  | 277.2176 |  | | | | |
| SQDG(36:7) | 835.4666 | 835.4676 | **1.2** | 18:3-18:4 | 225.0075 | 277.2169- - |  | | | | |
|  |  |  |  | 20:4-16:3 |  | 303.2329 - - |  | | | | |
|  |  |  |  | 20:5-16:2 |  | 301.2162- - |  | | | | |
| SQDG(34:3-OH) | 831.4928 | 831.4930 | **0.2** | ** | 225.0073 | - |  | | | | |
| SQDG(34:4-OH) | 829.4772 | 829.4752 | **-2.4** | ** | 225.0072 | - |  | | | | |
| **SQMG identified as [M−H]^−^** | | | | | | | |  | |  | |
| SQMG(16:0) | 555.2839 | 555.2838 | **-0.2** | * | 225.0068 | - |  | | | | |

MGDG - monogalactosyldiacylglycerol. MGMG - monogalactosylmonoacylglycerol. DGDG- digalactosyldiacylglycerol. DGMG – digalactosylmonoacylglycerol. SQDG - sulfoquinovosyl diacylglycerol. SQMG - sulfoquinovosyl monoacylglycerol. LPC - lysophosphatidylcholine. PC - phosphatidylcholine. LPE - lysophosphatidylethanolamine. PE - phosphatidylethanolamine. PG – phosphatidylglycerol. PI – phosphatidylinositol. DGTS - diacylglycerol-trimethylhomoserine. MGTS - monoacylglycerol-trimethylhomoserine. PI-Cer – inositol phosphoceramide; C – number of carbon atoms; N – number of double bonds; * - identified based on the polar head fragmentation. calculated mass. and retention time; ** - identified according to the calculated mass and the retention time; - the fragment/neutral loss was not observed. Note: for DGTS. the fatty acyl composition was assigned by the deduction by the losses of fatty acyl chains as acid (-RCOOH). represented without italic. and keto (R=C=O). represented in italic. derivatives.

**Supplementary table 3 –** LC–MS/MS spectra of oxidized product ions observed for oxidized PG. SQDG and DGTS species. Identification of oxidized polar lipids were confirmed by the presence of RCOO^-^ ion of oxidized fatty acids and by product ions resulting from neutral lossof oxidized fatty acyl chains as acid (-RCOOH) or ketene (-R=C=O). All ions are assigned with their predicted formula. observed mass. calculated mass. and mass error.

| **[M−H]^−^ (*m/z*)** | **Oxidized Products ions [RCOO]^−^** | **Formula** | **Calculated mass (Da)** | **Observed mass (Da)** | **Error (ppm)** |
| --- | --- | --- | --- | --- | --- |
| **733.4656** | **Fom PG(32:2-OH)** |  |  |  |  |
|  | 16:0(OH) | C_16_H_31_O_3_ | 271.2273 | 271.2281 | -2.9 |
| **763.5125** | **Fom PG(34:1-OH)** |  |  |  |  |
|  | 18:0(OH) | C_18_H_35_O_3_ | 299.2586 | 299.2600 | -4.6 |
| **761.4969** | **Fom PG(34:2-OH)** |  |  |  |  |
|  | 18:0(OH) | C_18_H_35_O_3_ | 299.2586 | 299.2595 | -2.9 |
| **759.4812** | **Fom PG(34:3-OH)** |  |  |  |  |
|  | 18:0(OH) | C_18_H_35_O_3_ | 299.2586 | 299.2594 | -2.6 |
|  | 18:1(OH) | C_18_H_33_O_3_ | 297.2430 | 297.2437 | -2.5 |
|  | 18:2(OH) | C_18_H_31_O_3_ | 295.2273 | 295.2269 | 1.4 |
|  | 18:3(OH) | C_18_H_29_O_3_ | 293.2117 | 293.2127 | -3.5 |
| **757.4656** | **Fom PG(34:4-OH)** |  |  |  |  |
|  | 18:4(OH) | C_18_H_27_O_3_ | 291.1960 | 291.1951 | 3.2 |
|  | 18:3(OH) | C_18_H_29_O_3_ | 293.2117 | 293.2121 | -1.5 |
|  | 18:2(OH) | C_18_H_31_O_3_ | 295.2273 | 295.2276 | -0.9 |
| **755.4499** | **Fom PG(34:5-OH)** |  |  |  |  |
|  | 18:4(OH) | C_18_H_27_O_3_ | 291.1960 | 291.1961 | -0.3 |
|  | 18:3(OH) | C_18_H_29_O_3_ | 293.2117 | 293.2131 | -4.9 |
| **783.4812** | **Fom PG(36:5-OH)** |  |  |  |  |
|  | 16:0(OH) | C_16_H_31_O_3_ | 271.2273 | 271.2280 | -2.5 |
|  | 20:5(OH) | C_20_H_29_O_3_ | 317.2117 | 317.2121 | -1.4 |
| **781.4656** | **Fom PG(36:6-OH)** |  |  |  |  |
|  | 20:5(OH) | C_20_H_29_O_3_ | 317.2117 | 317.2122 | -1.7 |
| **831.4928** | **Fom SQDG(34:3-OH)** |  |  |  |  |
|  | - | - | - | - | - |
| **829.4772** | **Fom SQDG(34:4-OH)** |  |  |  |  |
|  | - | - | - | - | - |
| **[M+H]^+^ (*m/z*)** | **Neutral loss of fatty acyl chains as acid (-RCOOH) or ketene (-R=C=O) derivatives** | **Formula** | **Calculated mass (Da)** | **Observed mass (Da)** | **Error (ppm)** |
| **746.5571** | **Fom DGTS(34:5-OH) (-R=C=O)** |  |  |  |  |
|  | -(C16:4(OH)-H2O) | C_28_H_54_O_6_N | 500.3951 | 500.3944 | 1.4 |
|  | -(C16:1(OH)-H2O) | C_28_H_48_O_6_N | 494.3482 | 494.3464 | 3.6 |
|  | -(C16:0(OH)-H2O) | C_28_H_46_O_6_N | 492.3325 | 492.3341 | -3.2 |
| **742.5258** | **Fom DGTS(34:7-OH) (-RCOOH)** |  |  |  |  |
|  | -(C16:3(OH)) | C_28_H_46_O_5_N | 476.3376 | 476.3352 | 5.0 |
|  | -(C16:4(OH)) | C_28_H_48_O_5_N | 478.3532 | 478.3511 | 4.5 |

**(B) MS/MS 552,3458** [LPC(16:1)+CH_3_COO]^−^

[RCOO]^−^

**(A) MS/MS 494.4,3246**

[LPC(16:1)+H]^+^

16:1

[LPC(16:1)+H]^+^

**Supplementary Figure 1 –** (**A**) LC-MS/MS spectrum of [M+H]^+^ ion of LPC(16:1) at *m/z* 494.3246. (**B**) LC-MS/MS spectrum of the [M+CH_3_COO]^−^ ion at *m/z* 552.3458 of the LPC(16:1). Fragment ions characteristic of LPC class were highlighted with a circle.

**MS/MS 758.5699**

[PC(34:2)+H]^+^

[PC(34:2)+H]^+^

**(B) MS/MS 816.5755**

[PC(34:2)+CH_3_COO]^−^

[RCOO]^−^

18:1

[RCOO]^−^

16:1

[RCOO]^−^

16:0

[RCOO]^−^

18:2

**Supplementary Figure 2 –**. (**A**) LC-MS/MS spectrum of the [M+H]^+^ ion at *m/z* 758.5699 of the PC(34:2). (**B**) LC-MS/MS spectrum of the [M+CH_3_COO]^−^ ion at *m/z* 816.5755 of the PC(34:2). Fragment ions characteristic of PC class were highlighted with a circle.

**(A) MS/MS 480.3090**

[LPE(18:1)+H]^+^

-[C_2_H_8_NO_4_P]

(-141Da)

**(B) MS/MS 478.2934**

[LPE(18:1)−H]^−^

18:1

[RCOO]^−^

[LPE(18:1)+H]^+^

**Supplementary Figure 3 -** (**A**) LC-MS/MS spectrum of the [M+H]^+^ ion at *m/z* 480.3090 of the LPE(18:1). (**B**) LC-MS/MS spectrum of the [M−H]^−^ ion at *m/z* 478.2934 of the LPE(18:1). Fragment ions characteristic of LPE class were highlighted with a circle.

**(A) MS/MS 688.4917**

[PE(32:2)+H]^+^

−[C_2_H_8_NO_4_P]

(−141Da)

**(B) MS/MS 686.4761**

[PE(32:2)−H]^−^

16:1

[RCOO]^−^

**Supplementary Figure 4 –** (**A**) LC-MS/MS spectrum of the [M+H]^+^ ion at *m/z* 688.4917 of the PE(32:2). (**B**) LC-MS/MS spectrum of the [M−H]^−^ ion at *m/z* 686.4761 of the PE(32:2). Fragment ions characteristic of PE class were highlighted with a circle.

18:3

16:0

[RCOO]^−^

[RCOO]^−^

**MS/MS 743.4867**

[PG(34:3)-H]^−^

[PI(34:1)-H]^−^

**(B) MS/MS 835.5302**

[PI(34:1)-H]^−^

C16:0

C18:1

[RCOO]^−^

[RCOO]^−^

**Supplementary Figure 5 –** (**A**) LC-MS/MS spectrum of the [M−H]^−^ ion at *m/z* 743.4867 of the PG(34:3). (**B**) LC-MS/MS spectrum of the [M−H]^−^ ion at *m/z* 835.5302 of the PI(34:1). Fragment ions characteristic of PG and PI class were highlighted with a circle.

16:1

[RCOO]^−^

**(A) MS/MS 757.4656**

[PG(34:4-OH)−H]^−^

C16:0

[RCOO]^−^

[RCOO]^−^

[RCOO] −

18:2-OH

18:4

[RCOO]^−-^

[RCOO]^−^

16:2

18:3-OH

18:4-OH

[RCOO-O]^−^

18:3

[RCOO-O]^−^

**Supplementary Figure 6 –** (**A**) LC-MS/MS spectrum of the [M−H]^−^ ion at *m/z* 757.4656 of the PG(34:4-OH). Water losses on the oxidized fatty acids were observed. Fragment ions characteristic of PG class were highlighted with a circle.

**(A) MS/MS 494.3482**

[MGTS(18:4)+H]^+^

[MGTS(18:4)+H]^+^

Neutral Loss of H_2_O

[C_10_H_22_O_5_N]^+^

= Neutral Loss of RCO

**(B) MS/MS 732.5778**

[DGTS(34:4)+H]^+^

[DGTS(34:4)+H]^+^

[C_10_H_22_O_5_N]^+^

Neutral Loss of RCO (18:4)

Neutral Loss of RCOOH (18:4)

Neutral Loss of RCO (16:0)

**Supplementary Figure 7 –** (**A**) LC-MS/MS spectrum of the [M+H]^+^ ion at *m/z* 494.3482 of the MGTS(18:4). (**B**) LC-MS/MS spectrum of the [M+H]^+^ ion at *m/z* 732.5778 of the DGTS(34:4).

**(A) MS/MS 730.5622**

[DGTS(34:5)+H]^+^

[DGTS(34:5)+H]^+^

[C_10_H_22_O_5_N]^+^

Neutral Loss of RCO (16:4)

**(B) MS/MS 746.5571**

[DGTS(34:5-OH)+H]^+^

[C_10_H_22_O_5_N]^+^

Neutral Loss of RCO (16:4-OH)

Neutral Loss of RCO (16:1-OH)

Neutral Loss of RCO (16:0-OH)

**Neutral Loss of H_2_O**

-32Da

**Supplementary Figure 8 –** (**A**) LC-MS/MS spectrum of the [M+H]^+^ ion at *m/z* 730.5622 of the DGTS(34:5). (**B**) LC-MS/MS spectrum of the [M+H]^+^ ion at *m/z* 746.5571 of DGTS(34:5-OH).

**(B) MS/MS 762.5156**

[MGDG(34:7)+NH_4_]^+^

**(A) MS/MS 536.3798**

[MGMG(18:1)+NH_4_]^+^

18:1

[RCO+74]^+^

[MGMG(18:1)+NH_4_]^+^

[RCO+74]^+^

[RCO+74]^+^

16:4

18:3

[M+NH_4_−197]^+^

**Supplementary Figure 9 –** (**A**) LC-MS/MS spectrum of the [M+NH_4_]^+^ ion at *m/z* 536.3798 of the MGMG(18:1). (**B**) LC-MS/MS spectrum of the [M+NH_4_]^+^ ion at *m/z* 762.5156 of the MGDG(34:4).

**(A) MS/MS 502.3016**

[MGMG(16:4)+NH_4_]^+^

**(B) MS/MS 518.2965**

[MGMG(16:4-OH)+NH_4_]^+^

16:4

[RCO+74]^+^

[MGMG(16:4)+NH_4_]^+^

16:4-OH

[RCO+74]^+^

[MGMG(16:4-OH)+NH_4_]^+^

**Supplementary Figure 10 –** (**A**) LC-MS/MS spectrum of the [M+NH_4_]^+^ ion at *m/z* 502.3016 of the MGMG(16:4). (**B**) LC-MS/MS spectrum of the [M+ NH_4_]^+^ ion at *m/z* 518.2965 of the MGMG(16:4-OH).

**(A) MS/MS 694.4014**

[DGMG(18:3)+NH_4_]^+^

18:3

[RCO+74]^+^

**(B) MS/MS 932.631**

[DGDG(34:3)+NH_4_]^+^

[DGDG(34:3)+NH_4_]^+^

16:0

[RCO+74]^+^

18:3

[RCO+74]^+^

18:1

[RCO+74]^+^

[M+NH_4_−359]^+^

**Supplementary Figure 11 –** (**A**) LC-MS/MS spectrum of the [M+NH_4_]^+^ ion at *m/z* 694.4014 of the DGMG(18:3). (**B**) LC-MS/MS spectrum of the [M+NH_4_]^+^ ion at *m/z* 932.631 of the DGDG(34:3).

[SQDG(32:0)+H]^−^

**(A) MS/MS 791.4979**

[SQDG(32:1)+H]^−^

C16:0

C16:1

[RCOO]^−^

[RCOO]^−^

**Supplementary Figure 12 –** (**A**) LC-MS/MS spectrum of the [M−H]^−^ ion at *m/z* 791.4979 of the SQDG(32:1). Fragment ions characteristic of SQDG class were highlighted with a circle.

[SQDG(34:3-OH)-H]^−^

C16:0

[RCOO]^−^

C18:3

[RCOO]^−^

C18:3-OH

[RCOO]^−^

**(A) MS/MS 831.4928**

[SQDG(34:3-OH)-H]^−^

**Supplementary Figure 13 –** (**A**) LC-MS/MS spectrum of the [M−H]^−^ ion at *m/*z 831.4928 of the SQDG(34:3-OH). Water losses on the oxidized fatty acids were observed. Fragment ions characteristic of SQDG class were highlighted with a circle.
